# Albumin binding improves the pharmacokinetics and therapeutic efficacy of a ^177^Lu-labeled HER2 Fab radioconjugate

**DOI:** 10.1126/sciadv.aee4052

**Published:** 2026-07-17

**Authors:** Peifei Liu, Yizhen Pang, Cuicui Li, Yuqi Hua, Wenyao Zhen, Tianzhi Zhao, Xiaobin Zhao, Jie Liu, Bingyu Li, Jinming Yu, Xiaoyuan Chen, Jingjing Zhang

**Affiliations:** ^1^ Center for Nuclear Medicine and Molecular Imaging, Shandong Cancer Hospital and Institute, Jinan 250117, China.; ^2^ Center for Molecular Imaging and Translational Medicine, School of Public Health, Xiamen University, Xiamen 361102, China.; ^3^ Department of Diagnostic Radiology, Yong Loo Lin School of Medicine, National University of Singapore, Singapore 119074, Singapore.; ^4^ Theranostics Center of Excellence, Yong Loo Lin School of Medicine, National University of Singapore, 11 Biopolis Way, Helios, Singapore 138667, Singapore.; ^5^ Clinical Imaging Research Centre, Centre for Translational Medicine, Yong Loo Lin School of Medicine, National University of Singapore, Singapore 117599, Singapore.; ^6^ Department of Nuclear Medicine, Affiliated Hospital of Jiangnan University, No. 1000, Hefeng Road, Wuxi 214028, China.; ^7^ Department of Nuclear Medicine, Beijing Tiantan Hospital, Capital Medical University, Nan Si Huan Xi Lu 119, Fengtai District, Beijing 100070, China.; ^8^ College of Basic Medicine and Forensic Medicine, Henan University of Science and Technology, Luoyang, Henan, China.; ^9^ Department of Radiation Oncology, Shandong Cancer Hospital and Institute, Shandong First Medical University and Shandong Academy of Medical Sciences, Jinan 250117, Shandong, China.

## Abstract

Therapeutic antibodies and antibody-drug conjugates are limited by large molecular size, poor tumor penetration, and signaling-adaptive resistance. We introduce an engineering strategy for radiotherapeutic antibody fragments in which an albumin-binding domain (ABD) rebalances molecular size and pharmacokinetics. Using human epidermal growth factor receptor 2 (HER2) as a proof of concept, we developed ^177^Lu-DOTA-Fab-ABD, which integrates rapid tumor penetration with albumin-mediated extended circulation, improving tumor dosimetry while reducing off-target radiation. In HER2 tumor models, ^177^Lu-DOTA-Fab-ABD showed strong HER2 and albumin binding, reduced off-target retention, and substantially lower hematologic toxicity than full-length ^177^Lu-DOTA-pertuzumab while maintaining potent β-particle tumor control. It also overcame intrinsic trastuzumab resistance by bypassing receptor blockade and inducing DNA double-strand breaks and displayed mechanistic complementarity with trastuzumab. Overall, ^177^Lu-DOTA-Fab-ABD represents a generalizable framework for engineering fragment–based radiotherapeutics.

## Teaser

An albumin-binding HER2 Fab radioconjugate reprograms pharmacokinetics to enhance targeted β-radionuclide therapy.

## INTRODUCTION

Overexpression of human epidermal growth factor receptor 2 (HER2/*ERBB2*) serves as a hallmark of several solid tumors, notably breast, gastric, ovarian, and colorectal cancers. The clinical validation of HER2 as a therapeutic target has been evidenced by the remarkable success of monoclonal antibodies, including trastuzumab and pertuzumab (Pert), and the subsequent development of antibody-drug conjugates (ADCs), such as trastuzumab emtansine (T-DM1) and trastuzumab deruxtecan (T-DXd) (*1*–*3*). Despite these advances, HER2-targeted biologics face multifactorial limitations: Their large molecular size (150 kDa) constrains tumor penetration and prolongs systemic exposure, thereby reducing the therapeutic index and increasing off-target hematologic and hepatic toxicities (*4*–*8*). Meanwhile, intratumoral heterogeneity, epitope and expression variability, Fc-mediated off-target effects, and suboptimal payload release further narrow their clinical benefit. Intrinsic and acquired resistance remain major challenges, particularly in tumors with poor accessibility or downstream escape mechanisms, exemplified by the trastuzumab-resistant JIMT-1 model (*9*–*11*).

To address these limitations, smaller antibody derivatives such as antigen-binding fragments (Fabs) have gained attention for their superior tumor penetration and faster systemic clearance (*12*–*14*). Nevertheless, the lack of an Fc domain and rapid renal elimination reduces their intratumoral retention and therapeutic efficacy. Various strategies have been developed to prolong the circulation time of antibody fragments, including PEGylation, Fc fusion, and albumin-mediated strategies. Among these, conjugation with an albumin-binding domain (ABD) represents an efficient and straightforward approach as its reversible interaction with serum albumin increases systemic retention and tumor deposition while preserving antigen-binding fidelity (*15*). Compared with other half-life extension methods, ABD fusion is small (5 to 6 kDa), substantially smaller than typical PEGylation (10 to 40 kDa) or Fc fusion (50 kDa per chain). By interacting with serum albumin and leveraging FcRn-mediated recycling, it enables an extended circulation half-life while maintaining efficient tissue penetration. Moreover, because ABD is genetically encoded as a single fusion, the resulting molecule exhibits higher structural uniformity and improved lot-to-lot consistency compared with chemically modified counterparts such as PEGylation or albumin “hitchhiking.”

While ABD-fused antibody fragments have demonstrated promise in imaging and pharmacokinetic modulation, their therapeutic application in targeted radionuclide therapy (TRT) remains underexplored. In particular, systematic comparisons with clinically established HER2-targeted antibodies and ADCs are lacking (*16*, *17*). Given the growing interest in radionuclide drug conjugates (RDCs) as next-generation therapeutics, Fab-based RDCs represent a compelling strategy. Unlike diabody, nanobody, affibody, or scFv-based ABD constructs, which typically exhibit disproportionately high renal retention and are therefore more suitable for diagnostic imaging than therapy (*18*, *19*), Fab-ABD could achieve a favorable balance between tumor penetration, circulation half-life, and safety profile, which renders it an attractive scaffold for therapeutic radionuclide applications (*20*, *21*).

In this study, we developed an ABD-fused Fab fragment labeled with lutetium-177 (^177^Lu-DOTA-Fab-ABD) and systematically evaluated its physicochemical properties, HER2 binding, in vivo imaging, and therapeutic activity in CT26-hHER2 and JIMT-1 tumor models. Notably, our Fab was derived from Pert rather than trastuzumab: Pert engages domain II of the HER2 ectodomain, whereas trastuzumab targets domain IV. Because first-line HER2 therapeutics, including most clinically approved ADCs such as T-DM1 and T-DXd, are trastuzumab-based and primarily target domain IV of the HER2 ectodomain, a Pert-derived radioconjugate circumvents epitope competition with domain IV–binding agents. Under clinically relevant dosing conditions, it thus offers strong combinability potential, complementing standard-of-care therapies and expanding translational relevance in HER2-positive cancers (*22*). We further evaluated the antitumor efficacy of ^177^Lu-DOTA-Fab-ABD in comparison with trastuzumab and clinically established HER2-ADCs, as well as its therapeutic synergy with trastuzumab. Overall, ^177^Lu-DOTA-Fab-ABD exhibits rapid tumor penetration with optimized pharmacokinetics and shows a favorable safety profile under the tested regimens in our mouse models. These findings highlight its potential as a HER2-targeted radiopharmaceutical and offer a practical framework for advancing fragment-based radiotherapeutics toward clinical translation.

## RESULTS

### Engineering and radiolabeling of Fab-ABD conjugates

**Fig. 1.**
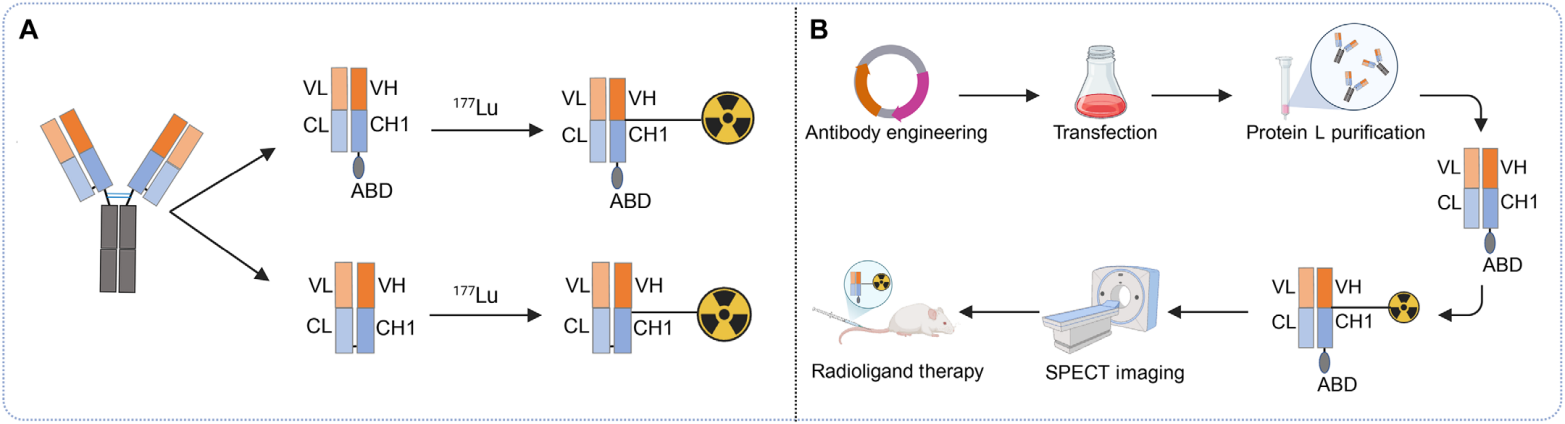
Engineering and radiolabeling of the albumin-binding Fab (Fab-ABD). (**A**) Schematic representation showing the construction of the Fab-ABD. A Pert-derived Fab was fused with an ABD at the C terminus of the heavy chain to generate a recombinant antibody fragment suitable for chelator conjugation. (**B**) DOTA conjugation and ^177^Lu radiolabeling of Fab-ABD and the control Fab (without ABD) to produce radioligands for SPECT imaging and radionuclide therapy studies. VL, variable light chain domain; VH, variable heavy chain domain; CL, constant light chain domain; CH, constant heavy chain domain. Created in BioRender. C. Ye (2026), https://BioRender.com/zoyvkhg.

We engineered a humanized HER2-specific Fab based on the variable regions of Pert, incorporating an ABD fused to the heavy-chain C terminus (Fig. 1A). Transient transfection in human embryonic kidney (HEK) 293 cells followed by protein L affinity chromatography yielded purified Fab-ABD suitable for conjugation. Fab-ABD and control Fab (lacking ABD) were conjugated with p-SCN-Bn-DOTA and radiolabeled with ^177^Lu under mild conditions, yielding radiochemical purities of >95% and high specific activities (figs. S1 to S4). This workflow generated radioligands for downstream in vitro binding assays, in vivo single-photon emission computed tomography (SPECT) imaging, and radionuclide therapy studies (Fig. 1B).

### In vitro validation of Fab-ABD albumin binding, HER2 targeting, and radioligand-induced DNA damage

**Fig. 2.**
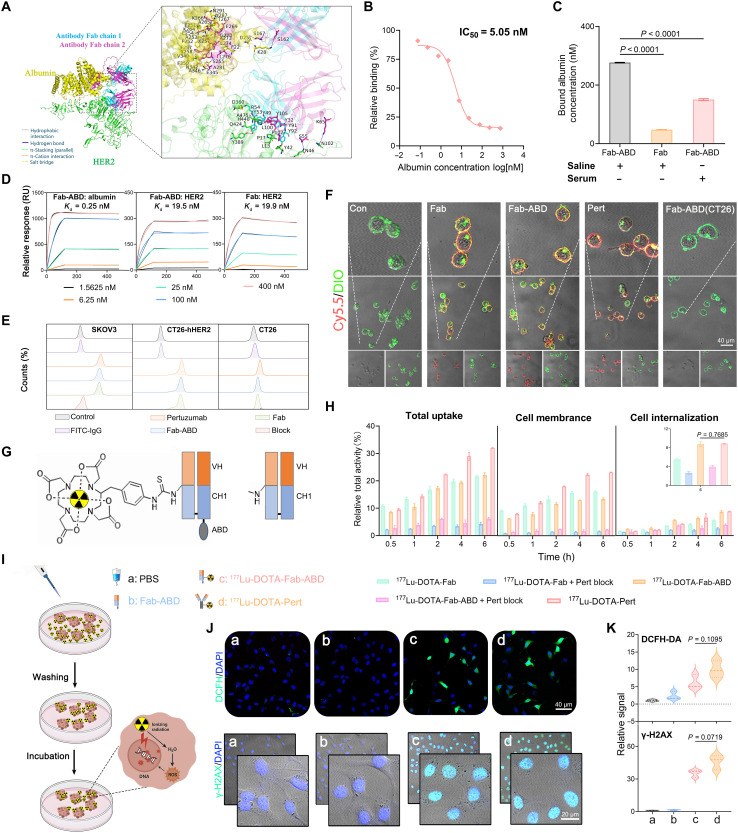
In vitro validation of Fab-ABD. (**A**) Structural model of the Fab-ABD complex bound to the HER2 ECD and human serum albumin (HSA), illustrating hydrogen bonding, hydrophobic interactions, and salt bridges. (**B**) Enzyme-linked immunosorbent assay (ELISA) quantifying albumin binding [median inhibitory concentration (IC_50_) of 5.05 nM]. (**C**) Competitive albumin-binding assay using mouse serum confirming ABD-mediated albumin interaction. (**D**) SPR analysis showing high-affinity albumin binding by Fab-ABD (*K*_d_ = 0.25 nM) and preserved HER2 affinity comparable to Fab and Pert. (**E**) Flow cytometry demonstrating HER2-specific binding of Fab-ABD and Fab, equivalent to Pert, with negligible binding to HER2-negative CT26 cells. (**F**) Confocal microscopy of Cy5.5-labeled antibodies showing membrane localization in CT26-hHER2 cells. The images are displayed together with the differential interference contrast (DIC) channel to illustrate cellular morphology and membrane localization (scale bar, 40 μm). The same confocal dataset is also presented in fig. S3, where the DIC channel is omitted to better visualize fluorescence signals. (**G**) Schematic of ^177^Lu labeling of Fab-ABD and Fab. (**H**) Cellular uptake and internalization kinetics (*n* = 4) showing comparable internalization of Fab-ABD and Pert at 6 hours. h, hours. (**I**) Schematic of the washed-cell protocol used before DNA damage assays. (**J**) Confocal imaging showing ROS (DCFH-DA; scale bar, 40 μm) production and DNA double-strand breaks (γ-H2AX; scale bar, 20 μm) after incubation with ^177^Lu-labeled constructs. DAPI, 4′,6-diamidino-2-phenylindole. (**K**) Quantification of ROS and γ-H2AX signals (*n* = 3), indicating no significant difference between Fab-ABD and Pert. Experiments were independently repeated with similar results. Data are presented as means ± SD; one-way analysis of variance (ANOVA).

We first validated the structural basis of Fab-ABD design. Molecular docking revealed that the HER2 extracellular domain (ECD)–Fab–ABD complex was stabilized by seven hydrophobic pairs, 20 hydrogen bonds, and four salt bridges, with a binding energy of −16.6 kcal mol^−1^ (fig. S5 and table S1). When Fab-ABD simultaneously engaged both HER2 ECD and serum albumin, the HER2 ECD-Fab-ABD interface remained stable, forming three hydrophobic pairs, eight hydrogen bonds, and one salt bridge [Gibbs free energy (Δ*G*) = −12.1 kcal mol^−1^], whereas the albumin-Fab-ABD interface comprised 14 hydrophobic pairs, 12 hydrogen bonds, and one salt bridge (Δ*G* = −9.6 kcal mol^−1^) (Fig. 2A). Although albumin association slightly attenuated the apparent binding affinity to HER2, this modest reduction is likely offset by the prolonged circulation time and enhanced systemic exposure conferred by albumin binding, thereby supporting effective tumor accumulation in vivo. These results suggest that Fab-ABD can concurrently recognize HER2 and albumin, with albumin binding exerting only a minor influence on HER2 interaction, providing a structural rationale for its dual-target design.

Experimental findings were consistent with the computational results. Enzyme-linked immunosorbent assay (ELISA) assays demonstrated that Fab-ABD bound albumin with high affinity [median inhibitory concentration (IC_50_) of 5.05 nM; Fig. 2B], whereas this interaction was significantly inhibited in the presence of mouse serum (*P* < 0.0001; Fig. 2C), confirming that the ABD domain mediates stable albumin binding under physiological conditions in vivo. Surface plasmon resonance (SPR) analysis confirmed subnanomolar affinity of Fab-ABD for albumin [dissociation constant (*K*_d_) = 0.25 nM] and HER2 binding that was comparable to both the parental Fab (*K*_d_ = 19.9 nM) and Pert (*K*_d_ = 21.4 nM), with Fab-ABD exhibiting a *K*_d_ = 19.5 nM (Fig. 2D, fig. S6, and table S2). Notably, human serum albumin (HSA) was used in the SPR analysis, whereas murine serum albumin was used in the ELISA experiments described above. These results indicate that the ABD domain incorporated in the Fab-ABD construct is capable of binding both murine serum albumin and HSA. An important advantage of this property is that it eliminates the need to replace or reengineer the ABD during clinical translation, thereby ensuring consistency between preclinical animal studies and potential human applications and enhancing the translational relevance of this platform. Collectively, these results demonstrate that ABD fusion markedly enhances albumin interaction and circulatory retention potential without compromising antigen recognition.

At the cellular level, flow cytometric analysis revealed that Fab-ABD and Fab bound HER2-positive SKOV3 and CT26-hHER2 cells with comparable efficiency to Pert, whereas binding to HER2-negative CT26 cells was negligible (Fig. 2E). Competitive inhibition confirmed that Pert completely blocked Fab-ABD binding, indicating that both recognize the same HER2 epitope. Confocal microscopy further verified that Cyanine 5.5 (Cy5.5)–labeled Fab-ABD, Fab, and Pert colocalized with the plasma-membrane dye 3,3′-dioctadecy (DIO), indicating specific recognition of membrane-bound HER2 (Fig. 2F and fig. S7). Quantitative fluorescence analysis (*n* = 3) showed no significant difference between Fab-ABD and Pert (*P* = 0.3229; fig. S8), suggesting that Fab-ABD largely retains the cellular binding affinity of the full-length antibody.

We then conjugated p-SCN-Bn-DOTA to Pert for ^177^Lu labeling (figs. S1 and S9D), achieving a radiochemical purity of >95% (fig. S2). In parallel, Fab and Fab-ABD were modified to generate DOTA-Fab and DOTA-Fab-ABD (fig. S9, A and B). Competitive binding assays showed that DOTA conjugation did not affect HER2 affinity, with IC_50_ values of 6.38 nM (DOTA-Fab) and 6.44 nM (DOTA-Fab-ABD) in SKOV3 cells, and 8.90 and 5.03 nM, respectively, in CT26-hHER2 cells (fig. S10). Both constructs were subsequently radiolabeled with ^177^Lu (Fig. 2G and figs. S3 and S4), and ^177^Lu-DOTA-Fab-ABD exhibited good stability over the tested time period in both phosphate-buffered saline (PBS) and fetal bovine serum (FBS; fig. S11). Time-dependent uptake assays in SKOV3 cells revealed progressive accumulation of all constructs, reaching 21.66 ± 0.40% for Fab and 22.15 ± 0.73% for Fab-ABD at 6 hours, with no notable difference, indicating that ABD fusion does not affect cellular uptake efficiency (Fig. 2H). Pert competition markedly reduced uptake to 4.28 ± 0.43% and 6.17 ± 0.38%, confirming that both Fab and Fab-ABD bind the same HER2 epitope as Pert but are distinct from that of trastuzumab (*22*, *23*). Internalization assays showed similar internalized fractions for Fab-ABD (8.78 ± 0.41%) and Pert (8.85 ± 0.10%) (*P* = 0.7685) but a higher internalization ratio for Fab-ABD (39.60 ± 0.79% versus 27.61 ± 0.24%, *P* = 0.0002), suggesting more efficient endocytosis per bound molecule (Fig. 2H and fig. S12). Comparable results were observed in CT26-hHER2 cells (fig. S13).

In HER2-negative CT26 cells, overall uptake remained low, with ^177^Lu-DOTA-Fab-ABD showing minimal uptake, whereas ^177^Lu-DOTA-Pert displayed relatively higher total uptake (10.38 ± 0.39%) and internalization (6.87 ± 0.59%) (fig. S14). These findings suggest that full-length Pert may undergo non–HER2-dependent internalization, possibly through Fc-mediated or nonspecific interactions (*24*), which could lead to off-target organ accumulation and increased radiation exposure in vivo, thereby constraining the therapeutic window and elevating toxicity risk.

Last, we assessed the radiocytotoxic effect of ^177^Lu-DOTA-Fab-ABD (Fig. 2I). SKOV3 cells treated with PBS (a), nonradiolabeled Fab-ABD (b), ^177^Lu-DOTA-Fab-ABD (c), or ^177^Lu-DOTA-Pert (d) exhibited markedly elevated intracellular reactive oxygen species (ROS) levels, as detected by 2′,7′-dichlorodihydrofluorescein diacetate (DCFH-DA) fluorescence (Fig. 2J, top), together with strong nuclear γ-histone H2A.X (γ-H2AX) staining indicative of radiation-induced DNA double-strand breaks (Fig. 2J, bottom). Quantitative analysis revealed slightly higher ROS and γ-H2AX signals in the ^177^Lu-DOTA-Pert group than in the ^177^Lu-DOTA-Fab-ABD group, although the differences were not statistically significant (ROS, *P* = 0.1095; γ-H2AX, *P* = 0.0719; *n* = 3; Fig. 2K). Together, these data demonstrate that ^177^Lu-DOTA-Fab-ABD efficiently enters HER2-positive cells, induces oxidative stress and DNA damage comparable to the full-length antibody, and maintains potent radiocytotoxic effect while offering advantages of smaller molecular size and reduced off-target uptake, thus providing both structural and functional justification for its potential in TRT (*25*).

### In vivo pharmacokinetics, biodistribution, and dosimetry of ^177^Lu-labeled constructs

**Fig. 3.**
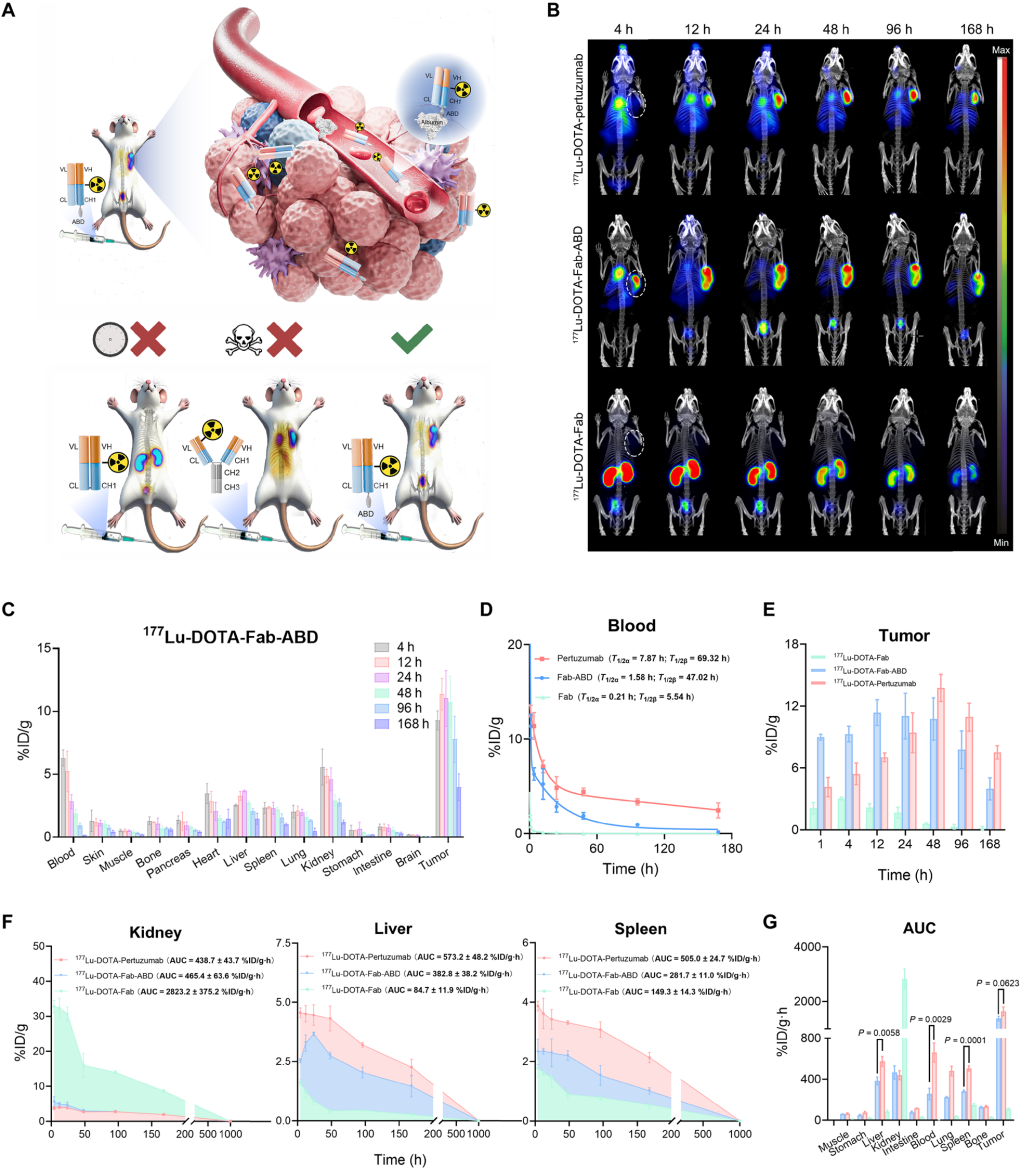
In vivo pharmacokinetics, biodistribution, and dosimetry. (**A**) Schematic representation of the in vivo comparison among ^177^Lu-DOTA-Pert, ^177^Lu-DOTA-Fab-ABD, and ^177^Lu-DOTA-Fab in CT26-hHER2 tumor–bearing mice. The schematic illustration in (A) was created using Adobe Illustrator. (**B**) Serial SPECT/CT imaging (4 to 168 hours) showing rapid and intense tumor visualization for Fab-ABD, delayed uptake for Pert, and weak tumor signal with fast renal clearance for Fab. (**C**) Biodistribution of ^177^Lu-DOTA-Fab-ABD (*n* = 3); biodistribution of Pert and Fab is provided in fig. S15 (A and B). (**D**) Blood pharmacokinetics fitted to a biexponential model: Pert, *T*_1/2__α_ = 7.87 hours and *T*_1/2__β_ = 69.32 hours; Fab-ABD, *T*_1/2__α_ = 1.58 hours and *T*_1/2__β_ = 47.02 hours; Fab, *T*_1/2__α_ = 0.21 hours and *T*_1/2__β_ = 5.54 hours. (**E**) Tumor uptake kinetics showing Fab-ABD peaking at 12 hours and surpassing Pert before 48 hours, whereas Fab remained low throughout (*n* = 3). (**F**) Organ dosimetry (*n* = 3). Area-under-the-curve (AUC) values are expressed as %ID/g· hour. Kidney AUC: Fab, 2823.2 ± 375.2; Pert, 438.7 ± 43.7; Fab-ABD, 465.4 ± 63.6. Liver exposure was significantly higher for Pert than for Fab-ABD (*P* = 0.0058). In the spleen, AUC was higher for Pert (505.0 ± 24.7) than for Fab-ABD (281.7 ± 11.0) (*P* = 0.0001). (**G**) Cumulative AUCs showing slightly higher tumor dose for Pert than Fab-ABD (*P* = 0.0623). Blood AUC was significantly lower for Fab-ABD than Pert (*P* = 0.0029), indicating reduced hematologic exposure, preserved hematopoietic and lymphoid compartments, and lower exposure in other organs. Data are presented as means ± SD; one-way ANOVA. h, hours.

In this study, we compared the behavior of ^177^Lu-DOTA-Pert, ^177^Lu-DOTA-Fab-ABD, and ^177^Lu-DOTA-Fab in CT26-hHER2 tumor–bearing mice (Fig. 3A). The schematic highlights three constructs: full-length immunoglobulin G1 (IgG1) with an Fc domain (Pert), a Fab fused to an ABD, and an unfused Fab, anticipated to display distinct distribution and clearance properties: prolonged circulation and higher reticuloendothelial system retention for Pert, rapid renal elimination for Fab, and an albumin-assisted intermediate profile for Fab-ABD that should enable earlier tumor access with reduced off-target retention. Serial SPECT/computed tomography (CT) imaging (Fig. 3B) confirmed this design logic: Fab-ABD afforded rapid, clear tumor visualization from 12 hours, Pert accumulated more slowly and peaked at 48 hours, and Fab produced a weaker tumor signal, consistent with fast renal clearance (fig. S15, A and B). The biodistribution of Fab-ABD verified early tumor uptake with minimal off-target accumulation (*n* = 3; Fig. 3C). In addition, to obtain a molecule with higher apparent affinity than Fab-ABD, we also designed a bivalent construct (Fab2-ABD). However, the in vivo results showed that, compared with ^177^Lu-DOTA-Fab-ABD, ^177^Lu-DOTA-Fab2-ABD exhibited lower tumor uptake at early time points (4 and 12 hours postinjection) and substantially higher liver and spleen uptake (fig. S15C). The increased molecular size reduced tumor penetration and altered clearance kinetics, suggesting enhanced accumulation in reticuloendothelial organs, thereby diminishing some of the intrinsic advantages of fragment-based antibody formats. Blood pharmacokinetics yielded distribution/elimination half-lives of 7.87/69.32 hours (Pert), 1.58/47.02 hours (Fab-ABD), and 0.21/5.54 hours (Fab), placing Fab-ABD between the full antibody and the fragment (Fig. 3D). Tumor uptake kinetics showed Fab-ABD peaking at 12 hours and surpassing Pert before 48 hours, whereas Fab remained low throughout (*n* = 4; Fig. 3E).

Dosimetry analyses revealed organ-specific dose differences (Fig. 3F). Kidney area under the curve (AUC) was highest for Fab (2823.2 ± 375.2) and much lower for Pert (438.7 ± 43.7) and Fab-ABD (465.4 ± 63.6), consistent with renal clearance of the unfused fragment. In contrast, hepatic and splenic exposures were significantly higher for Pert than for Fab-ABD (liver: 573.2 ± 48.2 versus 382.8 ± 38.2, *P* = 0.0058; spleen AUC: 505.0 ± 24.7 versus 281.7 ± 11.0, *P* = 0.0001). Aggregate AUCs indicated a slightly higher tumor dose for Pert compared with Fab-ABD, although the difference was not statistically significant (*P* = 0.0623) (Fig. 3G). Notably, the blood AUC of Fab-ABD was markedly lower than that of Pert (256.1 ± 55.2 versus 659.9 ± 92.6, *P* = 0.0029), indicating a reduced systemic radiation burden; Fab-ABD also showed markedly lower exposure in nontarget organs including the lung (223.8 ± 6.8 versus 479.9 ± 45.7), intestine (76.8 ± 10.3 versus 115.0 ± 2.4), and stomach (48.8 ± 6.8 versus 74.8 ± 10.4), collectively supporting a more favorable pharmacokinetic and safety profile (fig. S16).

Additionally, PET imaging with ^89^Zr-DFO-Fab-ABD also showed rapid and sustained tumor localization in CT26-hHER2 xenografts (fig. S17), with signal distribution closely matching the pharmacokinetic profile observed in the ^177^Lu-DOTA-Fab-ABD SPECT studies. This consistency across imaging modalities confirms the robust and rapid HER2 targeting of the Fab-ABD platform. Notably, the prolonged tumor retention and high tumor-to-background contrast highlight the potential of Fab-ABD–based tracers not only for radionuclide therapy but also for clinical HER2-directed PET diagnosis and longitudinal imaging.

### Therapeutic efficacy and hematologic safety of ^177^Lu-DOTA-Fab-ABD in CT26-hHER2 tumors

**Fig. 4.**
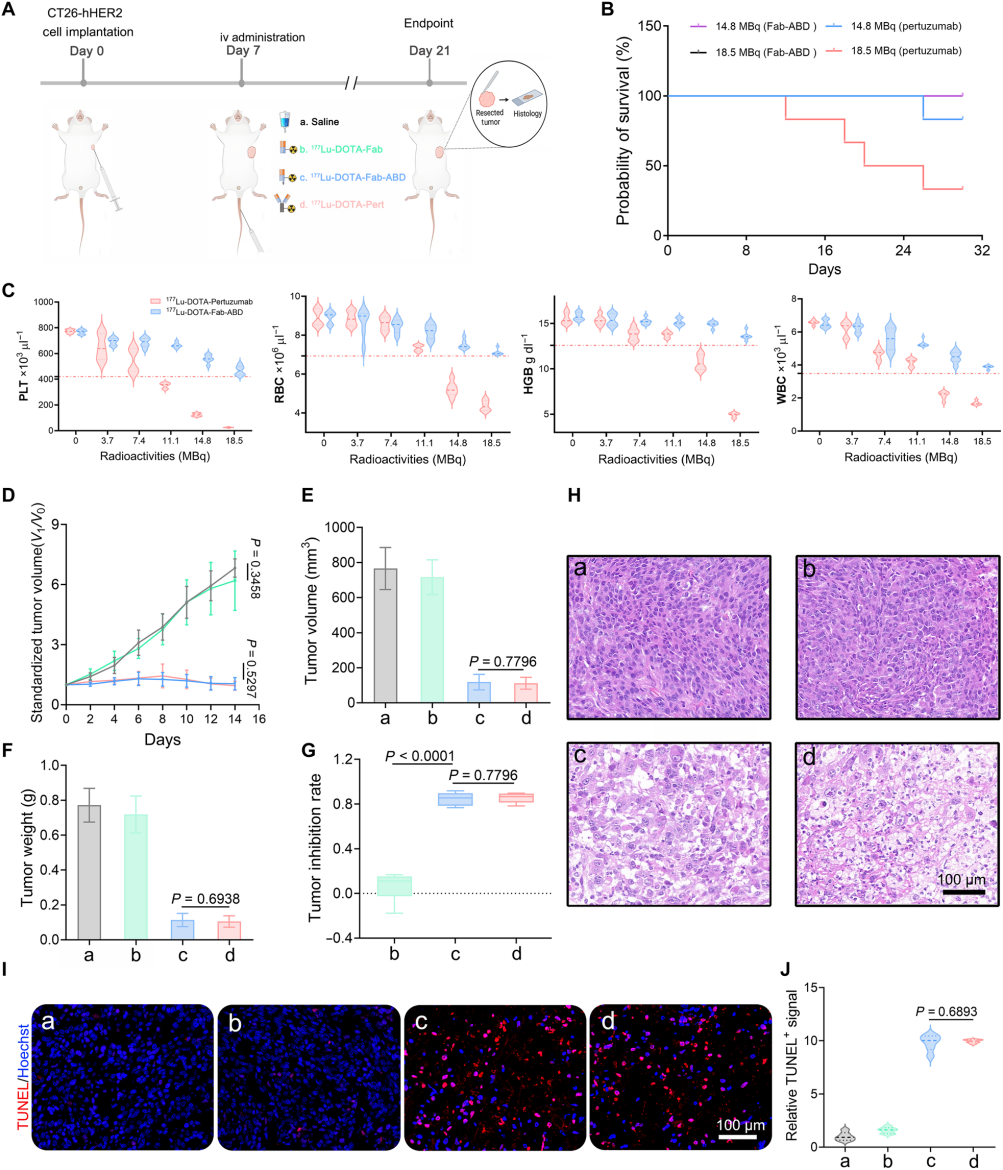
Therapeutic efficacy and hematologic safety of ^177^Lu-DOTA-Fab-ABD. (**A**) Study scheme for treatment groups: saline, ^177^Lu-DOTA-Fab, ^177^Lu-DOTA-Fab-ABD, and ^177^Lu-DOTA-Pert, in CT26-hHER2 tumor–bearing mice (*n* = 6 per group). iv, intravenous. (**B**) Kaplan-Meier survival curves at 14.8 and 18.5 MBq showing 100% survival for Fab-ABD and dose-dependent mortality for Pert (14.8 MBq, one of six deaths; 18.5 MBq, four of six deaths). (**C**) Complete blood counts (CBCs) after escalating activities (0 to 18.5 MBq) demonstrating overall higher platelet (PLT), red blood cell (RBC), hemoglobin (HGB), white blood cell (WBC), and lymphocyte (LYM) levels with Fab-ABD than with Pert; RBC, WBC, PLT, and HGB remained within physiological ranges (red dashed lines). (**D**) Tumor growth curves. (**E**) Endpoint tumor volumes showing no significant difference between Fab-ABD and Pert (117.97 ± 40.53 versus 111.42 ± 30.93 mm^3^; *P* = 0.7796). (**F**) Endpoint tumor weights showing no significant difference between Fab-ABD and Pert (*P* = 0.6938). (**G**) Tumor inhibition rates indicating Fab-ABD > Fab (*P* < 0.0001) and Fab-ABD ≈ Pert (*P* = 0.7796). (**H**) Hematoxylin and eosin (H&E) staining of excised tumors (scale bar, 100 μm). (**I**) TUNEL staining showing apoptosis in Fab-ABD and Pert groups (scale bar, 100 μm). (**J**) Quantification of TUNEL signals showing no significant difference between Fab-ABD and Pert (*P* = 0.6893). Data are presented as means ± SD; one-way ANOVA.

To evaluate the in vivo therapeutic efficacy and safety of the radioconjugates, we designed a single-dose treatment study in CT26-hHER2 tumor–bearing BALB/c mice (Fig. 4A). The schematic outlines the experimental workflow: tumor cell implantation on day 0, followed by intravenous administration of saline, ^177^Lu-DOTA-Fab, ^177^Lu-DOTA-Fab-ABD, or ^177^Lu-DOTA-pert on day 7, and endpoint analysis on day 21, including tumor excision and histopathological evaluation. Before therapy, a dose-escalation safety study was performed in healthy mice to determine the tolerable activity range [3.7 to 18.5 megabecquerels (MBq)]. Kaplan-Meier survival analysis and body-weight monitoring showed full survival and stable body weight for Fab-ABD up to 18.5 MBq, whereas Pert yielded 83.3% survival at 14.8 MBq (one of six deaths) and 33.3% at 18.5 MBq (four of six deaths), indicating dose-dependent toxicity (Fig. 4B and fig. S18). Complete blood counts (CBCs) across escalating doses revealed that ^177^Lu-DOTA-Fab-ABD maintained white blood cell (WBC), red blood cell (RBC), platelet (PLT), hemoglobin (HGB), and lymphocyte (LYM) counts largely within or above the physiological thresholds (red dashed lines), even at the highest tested activity of 18.5 MBq (Fig. 4C, table S3, and fig. S19). In contrast, ^177^Lu-DOTA-pert caused a dose-dependent decline in all five parameters, with values falling below the normal range at ≥11.1 MBq, indicative of myelosuppression (*26*). The inclusion of the red dashed line highlights this difference, illustrating that Fab-ABD treatment preserved hematologic stability across the tested dose range, whereas Pert-based radioconjugate induced hematologic toxicity in a dose-dependent manner.

Subsequently, CT26-hHER2 tumor–bearing mice were treated with a single injection of saline, ^177^Lu-DOTA-Fab, ^177^Lu-DOTA-Fab-ABD, or ^177^Lu-DOTA-Pert (*n* = 6 per group). Both ^177^Lu-DOTA-Fab-ABD and ^177^Lu-DOTA-pert notably suppressed tumor growth compared with the saline group (fig. S20). Quantitatively, the difference between the two radioconjugates was not statistically significant (*P* = 0.5297), suggesting comparable therapeutic efficacy. In contrast, ^177^Lu-DOTA-Fab exhibited only modest tumor control, without reaching statistical significance relative to the control group (*P* = 0.3458; Fig. 4D). Endpoint tumor volumes were comparable between Fab-ABD and Pert (117.97 ± 40.53 versus 111.42 ± 30.93 mm^3^, *P* = 0.7796; Fig. 4E), as were tumor weights (*P* = 0.6938; Fig. 4F). Tumor inhibition rates confirmed that Fab-ABD significantly outperformed Fab (*P* < 0.0001) and was equivalent to Pert (*P* = 0.7796; Fig. 4G). Histological analysis revealed reduced tumor cell density and extensive necrosis in both Fab-ABD– and Pert-treated groups (Fig. 4H), whereas hematoxylin and eosin (H&E) staining of major organs showed no apparent treatment-related pathology (fig. S21). Terminal deoxynucleotidyl transferase–mediated deoxyuridine triphosphate nick end labeling (TUNEL) staining demonstrated pronounced apoptosis in Fab-ABD and Pert tumors, with quantitative analysis showing no significant difference between the two groups (*P* = 0.6893; Fig. 4, I and J), with preserved HER2 expression across all groups as confirmed by IHC (fig. S22). Collectively, these data demonstrate that ^177^Lu-DOTA-Fab-ABD achieves potent tumor growth inhibition comparable to full-length Pert while offering improved hematologic safety and reduced systemic toxicity, supporting its translational potential as a safer HER2-targeted radionuclide therapeutic.

### Combination with trastuzumab augments ^177^Lu-DOTA-Fab-ABD efficacy, and Fab-ABD–based RDC outperforms ADC

**Fig. 5.**
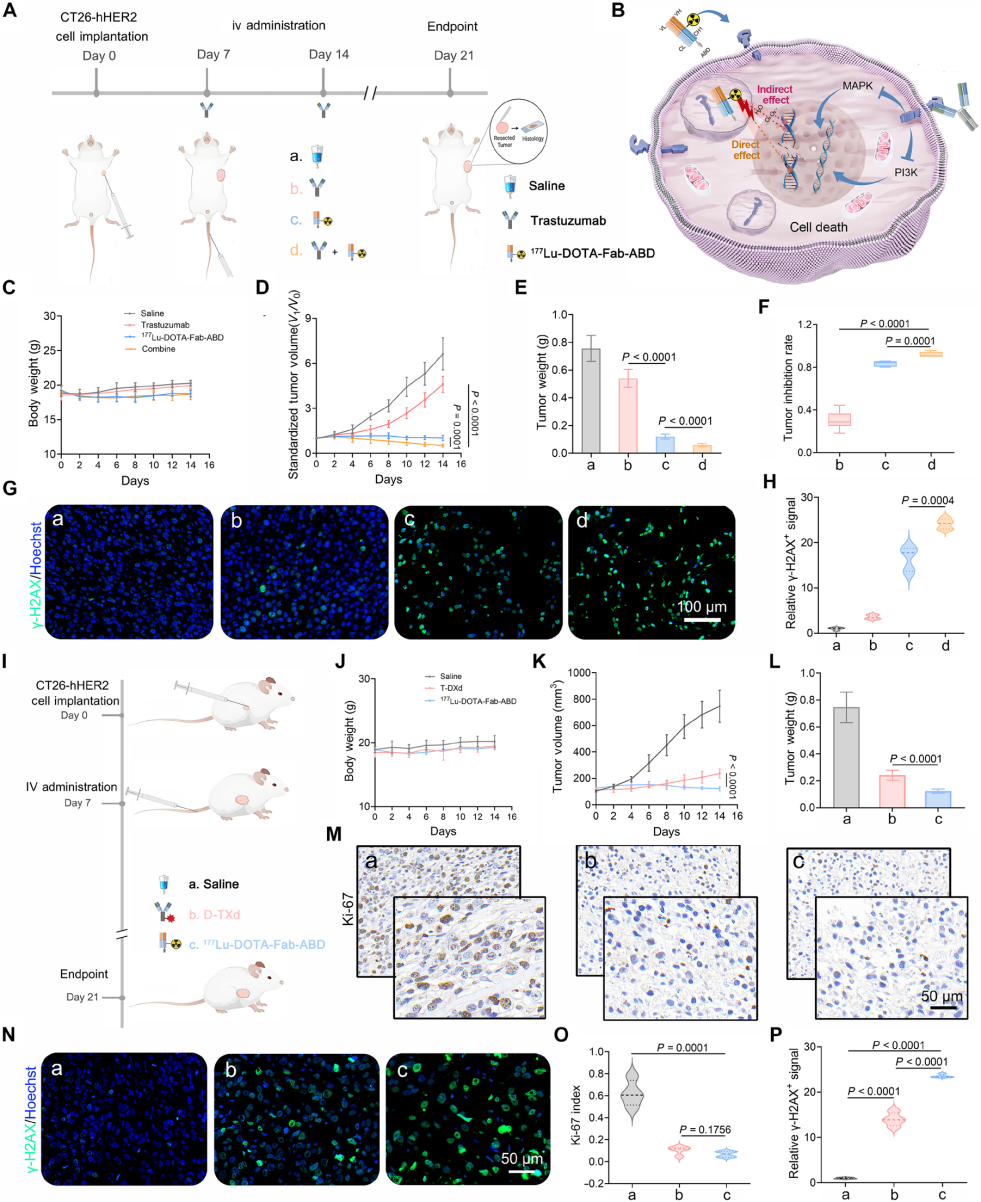
Combination therapy and comparative efficacy of Fab-ABD–based RDC versus ADC. (**A**) Treatment scheme. iv, intravenous. (**B**) Potential mechanistic overview showing that trastuzumab attenuates HER2 signaling and promotes receptor internalization, enhancing Fab-ABD surface engagement, whereas ^177^Lu-DOTA-Fab-ABD delivers β-radiation that directly induces nuclear DSBs; the combination converges on increased nuclear damage. MAPK, mitogen-activated protein kinase; PI3K, phosphatidylinositol 3-kinase. (**C**) Body-weight monitoring showing stable profiles across groups. (**D**) Tumor growth curves showing stronger suppression with the combination than with either monotherapy. (**E**) Endpoint tumor weight was lowest in the combination group (*P* < 0.0001 versus ^177^Lu-DOTA-Fab-ABD; *P* < 0.0001 versus trastuzumab). (**F**) Tumor inhibition rates: trastuzumab, 0.30 ± 0.07; ^177^Lu-DOTA-Fab-ABD, 0.84 ± 0.02; combination, 0.92 ± 0.02 (*P* < 0.0001 and *P* = 0.0001, respectively). (**G**) γ-H2AX immunofluorescence (IF; scale bar, 100 μm). (**H**) Quantification showing significantly higher DNA damage with the combination (*P* = 0.0004). (**I**) Schematic comparing the ADC (T-DXd) with the RDC (^177^Lu-DOTA-Fab-ABD). (**J**) Body-weight monitoring showing no major toxicity differences. (**K**) Tumor growth curves demonstrating stronger inhibition with the RDC. (**L**) Endpoint tumor weight was significantly lower with the RDC (*P* < 0.0001). (**M**) Ki-67 staining (scale bar, 50 μm). (**N**) γ-H2AX staining (scale bar, 50 μm). (**O**) Quantification showing no significant difference between ADC and RDC (*P* = 0.1756). (**P**) Quantification showing higher DNA damage with the RDC than with the ADC (*P* < 0.0001). Unless otherwise noted, data are means ± SD; one-way ANOVA with multiple comparisons.

Trastuzumab serves as the current first-line therapy for HER2-positive malignancies by recognizing domain IV of the HER2 extracellular region. In contrast, our ^177^Lu-DOTA-Fab-ABD targets domain II of HER2, providing a distinct epitope that enables concurrent administration with trastuzumab without competitive interference. There is also a theoretical potential for synergistic activity. Therefore, we evaluated the antitumor efficacy of combining trastuzumab with ^177^Lu-DOTA-Fab-ABD in tumor-bearing mice. CT26-hHER2 tumor–bearing mice were divided into four groups: saline, trastuzumab, ^177^Lu-DOTA-Fab-ABD, and the combination (Fig. 5, A to H). As shown in Fig. 5A, trastuzumab was administered intravenously on days 7 and 14, whereas ^177^Lu-DOTA-Fab-ABD was given as a single dose on day 7. The mechanistic illustration (Fig. 5B) depicts their synergistic actions: Trastuzumab attenuates HER2 signaling and promotes receptor internalization, thereby enhancing Fab-ABD surface engagement and endocytic uptake, whereas ^177^Lu-DOTA-Fab-ABD delivers β-particle radiation that directly induces nuclear DNA double-strand breaks. Together, these complementary mechanisms converge at the level of nuclear damage to amplify cytotoxicity. Body weights remained stable across all groups (Fig. 5C).

The control group in tumor growth curves (Fig. 5D and fig. S23) exhibited natural progression without intervention, whereas trastuzumab treatment slightly delayed tumor growth but showed only limited efficacy. This modest response is attributable to the biological characteristics of the murine CT26-hHER2 model, in which both human and endogenous murine HER2 pathways coexist (*26*). Because trastuzumab exclusively binds the human HER2 ECD IV, but not murine HER2, downstream signaling can be sustained through murine *Erbb2* and EGFR/HER3 heterodimers. Moreover, the Fc-mediated antibody-dependent cellular cytotoxicity (ADCC) activity of human IgG1 is limited in mice, collectively explaining the weak therapeutic effect of trastuzumab in this model.

By contrast, ^177^Lu-DOTA-Fab-ABD demonstrated significantly improved therapeutic efficacy, as evidenced by a marked reduction in tumor weight compared with trastuzumab monotherapy (Fig. 5E; *P* < 0.0001), which was further enhanced by combination with trastuzumab (*P* < 0.0001). Consistently, tumor volume analysis in fig. S24 showed that the combination group achieved the greatest tumor inhibition, with significantly lower tumor volume than the trastuzumab group (*P* < 0.0001) and the ^177^Lu-DOTA-Fab-ABD monotherapy group (*P* = 0.0001). In line with these findings, the combination group exhibited markedly slower tumor growth (Fig. 5D; *P* < 0.0001 and *P* = 0.0001, respectively), yielding the highest tumor-inhibition rate (0.92 ± 0.01; Fig. 5F; *P* < 0.0001 and *P* = 0.0001, respectively). The combination of ^177^Lu-DOTA-Fab-ABD and trastuzumab thus produced a synergistic antitumor effect, achieving marked tumor regression and superior inhibition compared with either agent alone. γ-H2AX staining confirmed DNA damage in all treated tumors, with the highest levels observed in the combination group, exceeding those in the ^177^Lu-DOTA-Fab-ABD group (Fig. 5, G and H; *P* = 0.0004). Consistent with this observation, trastuzumab pretreatment enhanced Fab-ABD binding and internalization in vitro (fig. S25), suggesting that dual-epitope engagement may promote HER2 receptor clustering and endocytosis, thereby increasing tumor uptake of the radioconjugate. Ki-67 staining further demonstrated reduced proliferative activity in the combination group (fig. S26) (*27*, *28*). In addition, hematological evaluation confirmed that ^177^Lu-DOTA-Fab-ABD, either alone or in combination with trastuzumab, maintained key peripheral blood parameters, including PLT, RBC, HGB, and WBC, within normal physiological ranges, comparable to controls (fig. S27). Combination therapy with the frontline HER2-targeted antibody enhanced antitumor efficacy without adding systemic or hematologic toxicity, further highlighting the excellent safety profile and clinical versatility of the ^177^Lu-DOTA-Fab-ABD platform. These findings underscore its potential for personalized and combinatorial HER2-targeted treatment strategies in clinical settings.

We next compared the Fab-ABD–based RDC (^177^Lu-DOTA-Fab-ABD) with the T-DXd (ADC). As shown in Fig. 5I, T-DXd was administered once intravenously on day 7. Body weights remained stable across groups (Fig. 5J). The RDC exhibited markedly greater tumor growth inhibition than the ADC (Fig. 5K; *P* < 0.0001), resulting in significantly lower endpoint tumor weights (Fig. 5L; *P* < 0.0001). Similar antitumor efficacy was also observed in the SKOV3 tumor model with endogenous HER2 expression (fig. S28). Consistent and pronounced tumor suppression was achieved following treatment with ^177^Lu-DOTA-Fab-ABD. Compared with both the saline and T-DXd groups, mice receiving ^177^Lu-DOTA-Fab-ABD exhibited marked and sustained inhibition of tumor growth. Quantitative analysis further demonstrated a statistically significant reduction in tumor volume in the ^177^Lu-DOTA-Fab-ABD group relative to that in the T-DXd group (*P* < 0.0001).

H&E staining revealed treatment-related necrosis in both groups, which was more extensive in RDC-treated tumors (fig. S29). Ki-67 immunohistochemistry (IHC) demonstrated that proliferative activity was significantly reduced in the RDC group compared with the saline control (*P* = 0.0001) and was slightly lower than in the ADC group, although the difference was not statistically significant (*P* = 0.1756; Fig. 5, M and O). In contrast, γ-H2AX quantification showed that RDC induced markedly stronger DNA damage than both saline and ADC treatments (all *P* < 0.0001), indicating more extensive β-particle–mediated double-strand breaks (Fig. 5, N and P). Hematological evaluation in healthy mice showed that both RDC and ADC maintained WBC, RBC, PLT, and HGB within physiological ranges, although T-DXd displayed a more evident downward trend, particularly in WBC, supporting the superior systemic safety profile of the Fab-ABD–based RDC (fig. S27) (*26*).

### ^177^Lu-DOTA-Fab-ABD overcomes trastuzumab resistance in JIMT-1 tumors

**Fig. 6.**
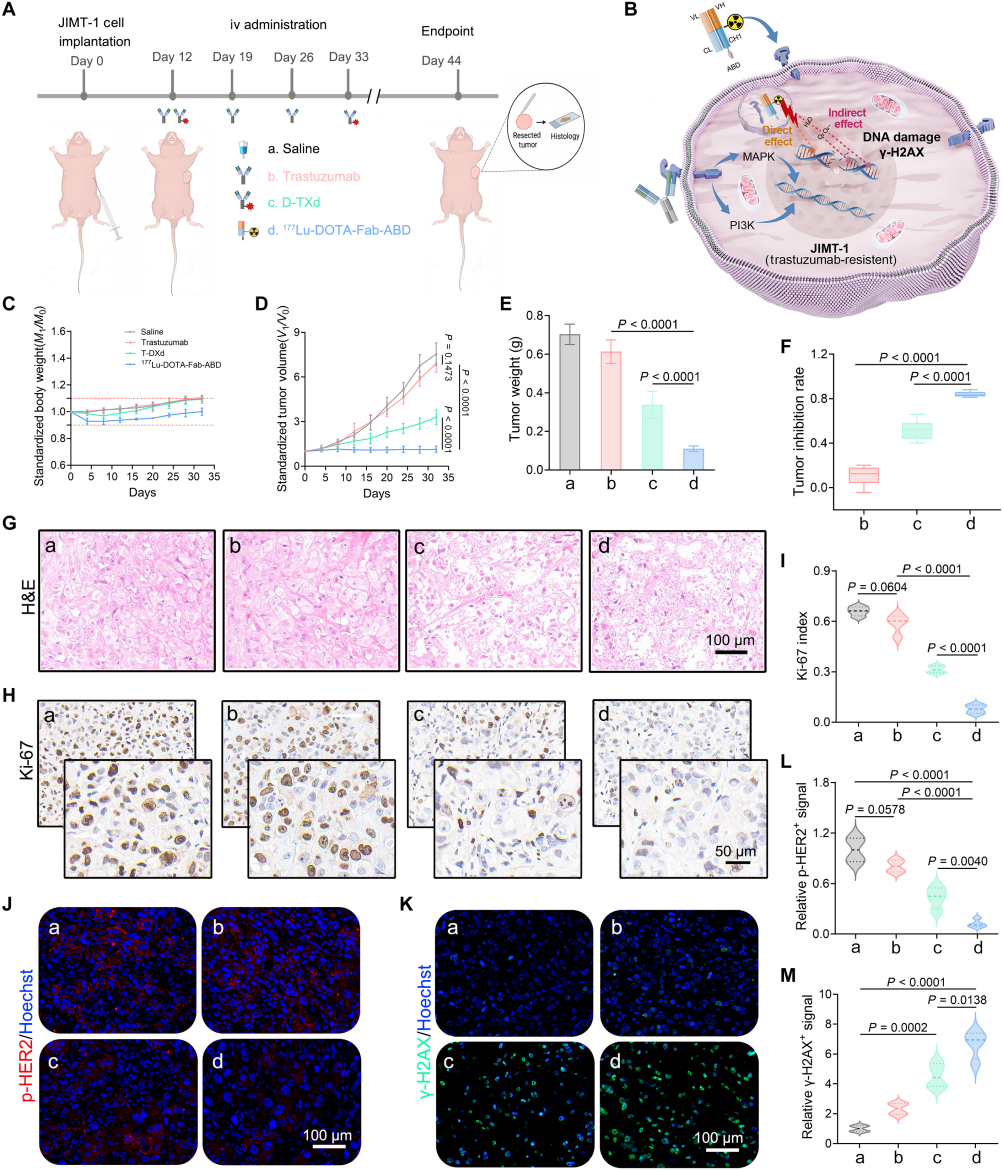
^177^Lu-DOTA-Fab-ABD overcomes trastuzumab resistance in JIMT-1 tumors. (**A**) Treatment scheme. (**B**) Potential mechanistic illustration showing that, in trastuzumab-resistant tumors, receptor blockade fails to suppress downstream HER2 signaling, whereas ^177^Lu-DOTA-Fab-ABD bypasses receptor signaling and delivers β-radiation that directly induces nuclear DSBs. (**C**) Body-weight monitoring (red dashed lines, ±10% of baseline). (**D**) Tumor growth curves. (**E**) Endpoint tumor weights were significantly lower with ^177^Lu-DOTA-Fab-ABD than with trastuzumab or ADC (*P* < 0.0001). (**F**) Tumor inhibition rates showing superior efficacy of ^177^Lu-DOTA-Fab-ABD over both comparators (*P* < 0.0001). (**G**) H&E staining (scale bar, 100 μm). (**H**) Ki-67 staining (scale bar, 50 μm). (**I**) Quantification showing reduced proliferation with ^177^Lu-DOTA-Fab-ABD. (**J**) p-HER2 IF (scale bar, 100 μm). (**K**) γ-H2AX staining (scale bar, 100 μm). (**L**) Quantification of relative p-HER2 fluorescence intensity across different treatment groups. (**M**) Quantification of relative γ-H2AX fluorescence intensity across different treatment groups. Unless otherwise noted, data are means ± SD; one-way ANOVA.

We next assessed efficacy in the trastuzumab-resistant JIMT-1 model. As shown in Fig. 6A, trastuzumab was administered intravenously on days 12, 19, and 26; T-DXd (5.4 or 10 mg/kg) was given on days 12, and 33, whereas a single dose of ^177^Lu-DOTA-Fab-ABD (RDC) was administered on day 12; Fig. 6B illustrates the potential mechanistic distinction, whereby trastuzumab fails to suppress downstream signaling in JIMT-1, whereas the RDC bypasses signaling to directly inflict β-particle–induced DNA damage. Body weights remained stable across groups (Fig. 6C and fig. S30). Tumor growth was markedly suppressed by the RDC compared with either trastuzumab or ADC monotherapy (Fig. 6D; both *P* < 0.0001), whereas the difference between trastuzumab and saline was not statistically significant (*P* = 0.1473; individual volumes in fig. S31). Even when the ADC dose was increased to 10 mg/kg, its antitumor activity improved but remained inferior to that of the RDC (*P* = 0.0093; fig. S32). The limited efficacy of T-DXd in the JIMT-1 model is consistent with known intrinsic resistance mechanisms, including PIK3CA mutations, E-cadherin loss, and high expression of drug efflux pumps (MDR1 and ABCG2), which collectively impair ADC internalization and payload release (*9*, *29*, *30*). Reduced lysosomal activity further diminishes T-DXd’s antitumor effect. In contrast, ^177^Lu-DOTA-Fab-ABD bypasses these resistance mechanisms and induces direct DNA damage through β-particle irradiation, thereby achieving superior tumor control (*31*, *32*). At study endpoint, tumor weight was significantly lower with the RDC than with either trastuzumab or ADC (*P* < 0.0001; Fig. 6E), and tumor-inhibition rate confirmed this superior activity (RDC versus trastuzumab, *P* < 0.0001; RDC versus ADC, *P* < 0.0001; Fig. 6F).

Histological and molecular analyses corroborated the enhanced antitumor activity of ^177^Lu-DOTA-Fab-ABD in the JIMT-1 model. H&E staining revealed extensive necrosis and structural disruption in RDC-treated tumors (Fig. 6G). Ki-67 IHC showed the lowest proliferative index in the RDC group (*P* < 0.0001 versus T-DXd; *P* < 0.0001 versus trastuzumab; Fig. 6, H and I), whereas the trastuzumab group retained abundant proliferating cells (*P* = 0.0604 versus saline). TUNEL staining further confirmed these findings, indicating enhanced apoptosis induced by radiation (fig. S33). These differences in tumor reflect the characteristic resistance of JIMT-1 cells, which maintain downstream HER2 signaling despite receptor blockade, thereby diminishing the efficacy of antibody and ADC therapies. Consistent with the underlying resistance mechanism, phosphorylated HER2 (p-HER2) immunofluorescence (IF) remained relatively strong in trastuzumab-treated tumors (*P* = 0.0578), whereas the ^177^Lu-DOTA-Fab-ABD group exhibited markedly reduced p-HER2 expression (*P* = 0.0040 versus T-DXd; *P* < 0.0001 versus trastuzumab; Fig. 6, J and L). Conversely, γ-H2AX staining revealed pronounced nuclear foci in both T-DXd and ^177^Lu-DOTA-Fab-ABD groups (*P* < 0.0001 versus saline for ^177^Lu-DOTA-Fab-ABD; *P* = 0.0002 for T-DXd; Fig. 6, K and M), with higher γ-H2AX intensity in ^177^Lu-DOTA-Fab-ABD than in T-DXd (*P* = 0.0138), suggesting more extensive β-particle–induced DNA double-strand breaks. Together, these findings indicate that ^177^Lu-DOTA-Fab-ABD circumvents receptor-signaling resistance through radiation-mediated DNA damage and apoptosis, achieving effective tumor control in HER2^+^ trastuzumab-resistant models.

In the JIMT-1 tumor–bearing mouse model, we further evaluated the dose-response relationship of ^177^Lu-DOTA-Fab-ABD using an activity gradient ranging from 3.7 to 33.3 MBq. At administered activities up to 18.5 MBq, body weight remained largely stable, whereas a clear downward trend was observed in the higher-activity groups (fig. S34A). Correspondingly, body weight showed a notable inverse correlation with administered activity (fig. S34B). Hematologic analysis revealed that both RBC and PLT gradually decreased with increasing activity (fig. S34, C and D). Among these parameters, PLT exhibited the highest sensitivity to radiation dose, displaying a stable linear decline with increasing activity [coefficient of determination (*R*^2^) = 0.8545, *P* < 0.0001]. Meanwhile, tumor inhibition increased markedly with administered activity and approached a plateau at ∼18.5 MBq (fig. S34E). Given that bone marrow suppression represents a principal dose-limiting toxicity in internal radionuclide therapy and that PLTs are generally more sensitive to radiation-induced injury, PLT was selected as the primary toxicity indicator for integrated efficacy-toxicity analysis (*33*). The analysis indicated that near-maximal tumor suppression could be achieved at around 18.5 MBq while PLT counts remained close to the physiological range; however, at higher activity levels, further gains in efficacy were limited, whereas PLT counts declined substantially (fig. S34F).

## DISCUSSION

This study establishes an albumin-binding Fab radioconjugate as a versatile platform for HER2-TRT, addressing key pharmacokinetic and therapeutic barriers associated with conventional antibodies and ADCs. Pharmacokinetic and biodistribution analyses demonstrated that ^177^Lu-DOTA-Fab-ABD achieved faster tumor uptake than ^177^Lu-DOTA-Pert while clearing more rapidly from nontarget tissues. Although peak tumor accumulation was slightly lower, dosimetric analysis confirmed comparable tumor-absorbed doses with markedly reduced radiation exposure to the liver, kidney, and spleen. This finding indicates that the modest attenuation in HER2 binding affinity upon albumin association does not compromise in vivo efficacy as it is effectively offset by prolonged circulation time and increased systemic exposure, leading to sufficient tumor dose deposition. Such redistribution is clinically meaningful, as antibody-based radiopharmaceuticals often display hepatosplenic or renal accumulation that constrains therapeutic index and leads to myelosuppression (*34*–*36*). The Fab-ABD construct thus achieves an optimal pharmacological balance between antibody fragments (nanobody, scFv, affibody, and diabody), which suffer from high renal retention (*37*), and full-length antibodies, which display excessive systemic exposure. The improved biodistribution and hematological tolerability observed in this study highlight Fab-ABD as a safer and more versatile scaffold for radionuclide therapy (*26*).

Therapeutically, ^177^Lu-DOTA-Fab-ABD demonstrated superior antitumor efficacy compared with trastuzumab and HER2-ADC in both CT26-hHER2 and trastuzumab-resistant JIMT-1 xenograft models. In JIMT-1 tumors, trastuzumab-treated samples retained high levels of p-HER2, indicating persistent HER2 signaling and therapeutic resistance. In contrast, ^177^Lu-DOTA-Fab-ABD treatment notably reduced p-HER2 levels, suggesting effective disruption of aberrant HER2-driven pathways. Prior studies have shown that radiation can promote HER2 dephosphorylation, disrupt membrane receptor clustering, and facilitate receptor internalization and degradation, ultimately attenuating HER2 kinase activity (*38*, *39*). In our study, γ-H2AX IF staining revealed extensive DNA double-strand breaks in tumors treated with Fab-ABD (RDC), exhibiting the highest fluorescence intensity among all groups (*P* < 0.0001), consistent with potent radiotoxic effects. Given the interplay between HER2 signaling and the DNA damage response, the observed γ-H2AX activation may further contribute to therapeutic efficacy by downregulating HER2 signaling through ATM/CHK2-mediated feedback inhibition (*40*, *41*). These findings highlight the multifaceted antitumor mechanism of ^177^Lu-DOTA-Fab-ABD and its potential to overcome resistance to conventional HER2-targeted therapies. Meanwhile, the favorable safety profile of Fab-ABD further supports its suitability for combination regimens without added systemic toxicity.

Combination therapy with ^177^Lu-DOTA-Fab-ABD and trastuzumab resulted in enhanced tumor suppression and increased γ-H2AX expression in CT26-hHER2 models, suggesting a synergistic antitumor mechanism. This synergy likely arises from dual epitope targeting of HER2, wherein ^177^Lu-DOTA-Fab-ABD delivers β-particle–mediated DNA damage, whereas trastuzumab inhibits HER2 signaling and promotes receptor internalization. The latter effect may enhance intratumoral retention of the radioconjugate, thereby amplifying its cytotoxicity. Such complementary actions are supported by prior studies showing that HER2 inhibition can sensitize tumor cells to radiotherapy by impairing DNA damage repair pathways and that the combination of trastuzumab with radiation can enhance DNA damage and therapeutic response in HER2-positive tumors (*38*, *42*, *43*). These findings support the translational potential of combining HER2-TRT with antibody-based blockade strategies, particularly in resistant disease contexts.

In summary, ^177^Lu-DOTA-Fab-ABD represents a promising HER2-targeted radiotherapeutic that integrates improved tissue penetration, reduced systemic toxicity, and potent antitumor efficacy, even in resistant models. By bridging antibody fragment engineering with radionuclide delivery, this strategy addresses fundamental limitations of conventional HER2-targeted therapies. Future studies should evaluate its performance in heterogeneous tumors, explore rational immunotherapy combinations, and optimize scaffold engineering to accelerate clinical translation.

## MATERIALS AND METHODS

### Ethics statement

All animal procedures were performed at the Laboratory Animal Center of Xiamen University and were approved by the Institutional Animal Care and Use Committee of Xiamen University (protocol XMULAC20200140). All procedures complied with institutional and national guidelines.

### Antibody fragment design, synthesis, and purification

Fab fragments, originating from the humanized monoclonal antibody Pert (Roche), were designed on the basis of its variable region sequences. These fragments were then produced using recombinant DNA techniques. To enhance pharmacokinetics, an ABD was genetically fused to the C terminus of the Fab, resulting in the creation of Fab-ABD constructs (*44*). These constructs were cloned into mammalian expression plasmids, specifically the PCDNA3.4 vector (Thermo Fisher Scientific), and subsequently transiently transfected into HEK293F cells (Thermo Fisher Scientific) to facilitate protein expression. The expressed proteins were purified using protein L affinity chromatography (Cytiva), followed by size exclusion chromatography (SEC; GE Healthcare). This two-step purification procedure yielded highly pure final protein products.

### Cell culture

Cell lines used in this study included SKOV-3, JIMT-1, and CT26 (American Type Culture Collection), as well as CT26-hHER2 stably transfected to overexpress the hHER2 (Nanjing CoBioer Biosciences, China). CT26 and CT26-hHER2 cells were cultured in RPMI 1640, SKOV-3 in McCoy’s 5A, and JIMT-1 in Dulbecco’s modified Eagle’s medium/F12 (1:1). Each medium was supplemented with 10% FBS (Invitrogen) and 1% penicillin-streptomycin, whereas JIMT-1 additionally required human insulin (10 μg/ml; Sigma-Aldrich). All cells were cultured at 37°C in a humidified 5% CO_2_ atmosphere and routinely tested to confirm the absence of mycoplasma contamination.

### SPR analysis

SPR experiments were performed on a Biacore T200 instrument (Cytiva) at 25°C. Recombinant human HER2 ECD (Sino Biological) and HSA (Sino Biological) were immobilized on separate flow cells of CM5 sensor chips using standard amine coupling, to a surface density of ∼200 response units (RU). Binding studies were conducted in HBS-EP^+^ buffer [10 mM Hepes, 150 mM NaCl, 3 mM EDTA, and 0.05% (v/v) surfactant P20 (pH 7.4)] at a flow rate of 30 μl min^−1^. Serial dilutions (1.56 to 400 nM) of full-length antibody, Fab, or Fab-ABD were injected over the immobilized surfaces with 180-s association and 300-s dissociation phases. The sensor surface was regenerated with 10 mM glycine-HCl (pH 2.5) for 30 s. For albumin-binding analysis, HSA was immobilized under identical conditions, and Fab-ABD was tested using the same concentration series and regeneration protocol. All sensorgrams were double referenced against blank injections and globally fitted to a 1:1 Langmuir binding model using Biacore T200 Evaluation Software.

### Flow cytometry

SKOV-3, CT26, and CT26-hHER2 cells were harvested and resuspended in cold PBS containing 1% bovine serum albumin (BSA) at a density of 1 × 10^6^ cells per 200-μl tube. Cells were incubated at 4°C for 1 hour with 10 μg ml^−1^ of fluorescein isothiocyanate (FITC)–labeled Pert, Fab, or Fab-ABD. Human IgG-FITC was used as a nonspecific control. For blocking experiments, FITC-Fab-ABD group was preincubated with a 100-fold molar excess of unlabeled Pert for 30 min at room temperature (RT) before cell incubation. After two washes with cold PBS, fluorescence signals were acquired using a Quanteon Express flow cytometer. Comparative binding among SKOV-3, CT26, and CT26-hHER2 cell lines was used to assess HER2 specificity, with CT26 (HER2^−^) serving as the negative control.

### Competitive ELISA to assess Fab-ABD binding to endogenous mouse serum albumin

To evaluate the binding specificity and affinity of Fab-ABD for HSA, a competitive ELISA was performed using a commercially available HSA-coated kit (Meilian Biotech, China). Fab-ABD (1 μg ml^−1^) was preincubated with serial dilutions of purified HSA (0 to 100 μg ml^−1^) for 30 min at RT and transferred to 96-well plates precoated with HSA. Optical density (at 450 nm) values were recorded, and inhibition curves were generated by plotting signal intensity against HSA concentration. The IC_50_ was determined by nonlinear regression using a four-parameter logistic model in GraphPad Prism.

To further confirm binding specificity, Fab-ABD and control Fab fragments (lacking ABD) were incubated with prediluted mouse serum (1:10 in PBS) or PBS alone. Bound Fab fragments were detected using biotinylated anti-Fab secondary antibodies followed by horseradish peroxidase–conjugated streptavidin, according to the manufacturer’s instructions. Absorbance was measured at 450 nm, and results were normalized to the positive control (Fab-ABD without competitor). All experiments were performed in triplicate, and data are presented as means ± SD.

### Cy5.5 labeling

Following buffer exchange into 0.1 M NaHCO_3_ (pH 9.2) using Amicon Ultra filters (10-kDa molecular weight cutoff, Merck Millipore), proteins were conjugated with Cy5.5–*N*-hydroxysuccinimide (NHS) ester (excitation/emission, 675/694 nm). The dye was added at a tenfold molar excess and incubated for 2 hours at RT. Labeled products were purified using PD-10 desalting columns (GE Healthcare) equilibrated with PBS (pH 7.4) to remove residual unbound fluorophore.

### IF staining for HER2 binding

CT26-hHER2 and wild-type CT26 cells were seeded onto confocal dishes and allowed to adhere overnight. Cells were fixed with 4% paraformaldehyde for 10 min at RT and blocked with 2% BSA for 30 min at 37°C. After blocking, cells were incubated for 30 min at 4°C in PBS containing Cy5.5-Fab, Cy5.5-Fab-ABD, or Cy5.5-Pert (10 μg ml^−1^); PBS alone served as the negative control. Plasma membranes were stained with the lipophilic dye DiO (Beyotime) for 15 min at 37°C, followed by three washes with warm PBS. Nuclei were counterstained with Hoechst 33342 (Beyotime) for 10 min. Fluorescence images were acquired on a Nikon A1RS confocal laser scanning microscope using identical acquisition settings across all groups.

### Conjugation, radiolabeling, and quality control

Antibody constructs, including Pert, Fab, and Fab-ABD, were conjugated with the bifunctional chelator p-SCN-Bn-DOTA (Macrocyclics, USA) according to previously reported protocols with slight modifications (*45*). Following buffer exchange into 0.1 M NaHCO_3_ (pH 9.2) using 10-kDa Amicon Ultra filters (Merck Millipore), proteins were reacted with a 20-fold molar excess of chelator for 2 hours at RT. The resulting conjugates were purified on PD-10 desalting columns (GE Healthcare) equilibrated with PBS (pH 7.4) to remove residual unbound chelators.

DOTA-protein conjugates were radiolabeled by reacting with ^177^LuCl_3_ (HTA Co., China) for 1 hour at 37°C in 0.1 M sodium acetate buffer (pH 5.5). Following purification through PD-10 columns, the labeling efficiency was analyzed by radio-thin-layer chromatography using silica gel plates and 50 mM citric acid/trisodium citrate (pH 5.0) as the eluent. Only samples achieving radiochemical purities above 95% were advanced to biological testing.

### In vitro stability

The in vitro stability of ^177^Lu-DOTA-Fab-ABD was evaluated in PBS (pH 7.4) and in PBS containing 10% FBS. The purified radioconjugate was incubated at 37°C under gentle agitation. Aliquots were collected at 0, 24, 72, 120, and 168 hours for radiochemical analysis. Radiochemical stability was analyzed using SEC radio-HPLC performed on a Shimadzu LC-20A system (Shimadzu, Japan) equipped with an inline radioactivity detector. Radiochemical purity was calculated as the percentage of radioactivity associated with the intact ^177^Lu-DOTA-Fab-ABD peak relative to the total detected radioactivity.

### Intracellular ROS generation

CT26-hHER2 cells were treated with PBS, Fab-ABD, ^177^Lu-DOTA-Fab-ABD (3.7 MBq ml^−1^), or ^177^Lu-DOTA-Pert (3.7 MBq ml^−1^) for 6 hours. Unbound probes were removed by 3× PBS washes, and cells were returned to probe-free complete medium for 24 hours. After incubation, cells were stained with 0.1 μM DCFH-DA (Beyotime) fluorescent probe for 30 min in a CO_2_ incubator. Nuclei were counterstained with Hoechst 33342 (Beyotime) for 10 min. Fluorescence images were acquired on a Nikon A1RS confocal laser scanning microscope using identical acquisition settings across all groups.

### Assessment of DNA damage via γ-H2AX IF

CT26-hHER2 cells were treated with PBS, Fab-ABD, ^177^Lu-DOTA-Fab-ABD (3.7 MBq ml^−1^), or ^177^Lu-DOTA-Pert (3.7 MBq ml^−1^) for 6 hours. Unbound probes were removed by 3× PBS washes, and cells were returned to probe-free complete medium for 24 hours. All subsequent steps were performed according to the manufacturer’s instructions (Beyotime kit C2037S) using ready-to-use reagents. Cells were fixed with the kit Fixative (C2037S-1, 10 min, RT), washed with Wash Buffer (C2037S-2), blocked with IF blocking buffer (C2037S-3, 30 min, 37°C), and incubated with mouse anti–γ-H2AX (Ser^139^) (C2037S-4, ready to use) for 1 hour at RT (or overnight at 4°C). After washing with Wash Buffer, cells were incubated with anti-mouse Alexa Fluor 488 secondary antibody (C2037S-5, ready to use) for 1 hour at RT in the dark, washed with Wash Buffer, and counterstained with 4′,6-diamidino-2-phenylindole (C2037S-6, 10 min). Last, samples were mounted with antifade mounting medium (C2037S-7). Fluorescence images were obtained using a Nikon A1RS confocal laser scanning microscope under identical acquisition settings across all groups.

### Systemic safety and dose-escalation study

The systemic safety and tolerability of ^177^Lu-DOTA-Fab-ABD and ^177^Lu-DOTA-Pert were evaluated in healthy BALB/c mice (6 to 8 weeks old, 18 to 22 g). All animal experiments were performed at the Laboratory Animal Center of Xiamen University and were approved by the Institutional Animal Care and Use Committee of Xiamen University Laboratory Animal Center (approval number XMULAC20200140). All procedures were conducted in accordance with the institutional and national ethical guidelines.

Radiolabeled constructs were intravenously administered via the tail vein at escalating doses of 3.7, 7.4, 11.1, 14.8, and 18.5 MBq (*n* = 6 per group), whereas control animals received PBS. Body weight was recorded every other day for 30 days postinjection as a general indicator of systemic toxicity, and survival was monitored daily. Survival data were analyzed using the Kaplan-Meier method.

To assess hematological toxicity, CBCs were collected from the retro-orbital sinus at day 14 after injection. Measured parameters included RBCs, WBCs, HGB, PLTs, and LYMs. Results were compared with reference physiological ranges reported for BALB/c mice (table S3).

### Cellular uptake, internalization, and binding affinity of radiolabeled constructs

To examine HER2-specific uptake and internalization of the ^177^Lu-labeled constructs, SKOV3, CT26-hHER2 (HER2^+^), and parental CT26 (HER2^−^) cells were plated in 24-well plates at 1.5 × 10^5^ cells per well and cultured overnight at 37°C. Before tracer exposure, the medium was replaced with serum-free medium after two PBS washes, followed by 1 hour of incubation at 37°C. For receptor-blocking assays, CT26-hHER2 cells were preincubated with a 100-fold molar excess of unlabeled Pert for 1 hour to occupy HER2 binding sites. Thereafter, cells were treated with ∼37 kBq (1 μCi) of ^177^Lu-DOTA-Fab, ^177^Lu-DOTA-Fab-ABD, or ^177^Lu-DOTA-Pert per well for 0.5, 1, 2, 4, or 6 hours at 37°C. Following incubation, unbound radioactivity was removed by two PBS washes, and cells were lysed in 200 μl of 0.1 M NaOH to quantify total associated activity using a γ-counter (PerkinElmer, Waltham, MA). Uptake was expressed as the percentage of the applied dose per well (%AD/well).

To distinguish membrane-bound and internalized fractions, surface-bound activity was removed using a mild acid wash (0.1 M glycine buffer, pH 2.5) for 5 min on ice, followed by PBS rinsing. The remaining counts represented the internalized fraction, and internalization efficiency was calculated as the ratio of internalized to total activity over time.

### Animal models

All animal experiments were performed at the Laboratory Animal Center of Xiamen University and were approved by the Institutional Animal Care and Use Committee of Xiamen University Laboratory Animal Center (approval number XMULAC20200140). All procedures were conducted in accordance with the institutional and national ethical guidelines. Female BALB/c mice (6 to 8 weeks old, 18 to 20 g) were subcutaneously injected in the right upper flank with 2 × 10^6^ CT26 or CT26-hHER2 cells suspended in 150 μl of PBS. Tumor dimensions were measured every 2 days using digital calipers, and volumes were calculated using the formula (length × width^2^)/2. For the human breast cancer model, female athymic nude mice (6 to 8 weeks old, 1 to 22 g) were subcutaneously inoculated in the right flank with 8 × 10^6^ JIMT-1 cells in a 1:1 mixture of PBS and Matrigel (Corning). For human ovarian cancer model, female athymic nude mice (6 to 8 weeks old, 18 to 22 g) were subcutaneously inoculated in the right flank with 1 × 10^7^ SKOV3 cells in a 1:1 mixture of PBS and Matrigel (Corning). Tumor dimensions were measured every 4 days using digital calipers, and volumes were calculated using the formula (length × width^2^)/2. In vivo imaging was initiated when CT26-hHER2 tumors reached ∼200 to 500 mm^3^, and mice were randomly assigned to treatment groups before intervention.

### In vivo small-animal SPECT/CT studies

To investigate the in vivo biodistribution and tumor-targeting performance of the ^177^Lu-labeled antibody constructs, small-animal SPECT/CT imaging was carried out using female BALB/c mice bearing subcutaneous CT26-hHER2 xenografts. Each mouse (*n* = 3 per group) received an intravenous injection of 7.4 MBq (200 μCi) of ^177^Lu-DOTA-Pert, ^177^Lu-DOTA-Fab-ABD, or ^177^Lu-DOTA-Fab in 100 μl of sterile saline. During image acquisition, anesthesia was maintained with 2% isoflurane in oxygen. Whole-body scans were obtained at 4, 12, 24, 48, 96, and 168 hours postinjection using a nanoScan SPECT/CT system (Mediso, Hungary). CT datasets were used for anatomical coregistration and attenuation correction.

SPECT and CT data were coregistered and reconstructed using Nucline software (Mediso), and fused images were generated to visualize tracer distribution. Identical acquisition parameters, reconstruction settings, and color scales were applied across all time points and experimental groups to ensure quantitative comparability.

### Biodistribution study

To assess the pharmacokinetic behavior and tissue distribution of the ^177^Lu-labeled antibody constructs, female BALB/c mice bearing CT26-hHER2 tumors received an intravenous injection of 0.74 MBq (20 μCi) of ^177^Lu-DOTA-Pert, ^177^Lu-DOTA-Fab-ABD, or ^177^Lu-DOTA-Fab in 100 μl of sterile saline. At scheduled intervals (1, 4, 12, 24, 48, 96, and 168 hours postinjection), three mice per group were euthanized, and samples of tumor, muscle, skin, bone, and major organs, including heart, liver, spleen, lungs, kidneys, intestines, stomach, pancreas, and brain, were excised, washed, weighed, and analyzed for radioactivity using a γ-counter (Wizard, PerkinElmer, Waltham, MA). Blood samples were collected at designated time points (1, 4, 12, 24, 48, 96, and 168 hours). Due to the rapid systemic clearance of the Fab fragment, an additional early time point (0.5 hours) was included for the Fab group to ensure accurate characterization of its initial pharmacokinetic profile.

The percentage of injected dose per gram of tissue (%ID g^−1^) was calculated by normalizing the measured radioactivity to the injected activity and tissue weight. Time-activity curves were plotted to characterize the distribution kinetics in tumors and normal organs. Pharmacokinetic parameters, including the distribution half-life (*T*_1/2α_) and elimination half-life (*T*_1/2β_), were determined by two-phase exponential fitting using DAS 2.0 software. The AUC was calculated for each organ to compare systemic exposure, clearance behavior, and tumor retention among the different radiolabeled constructs.

### Therapeutic efficacy study

Therapeutic studies were conducted using female BALB/c mice bearing subcutaneous CT26-hHER2 tumors and female athymic nude mice implanted with JIMT-1/SKOV3 xenografts. Once tumor volumes reached around 80 to 120 mm^3^, the animals were randomly divided into experimental groups (*n* = 6 per group) for treatment evaluation. Mice received a single intravenous injection of ^177^Lu-DOTA-Fab-ABD (11.1 MBq, 300 μCi) via the tail vein. For combination and comparison studies, trastuzumab [5 mg kg^−1^, intravenous (iv)] was administered once weekly (Q7D × 2; on days 7 and 14 for CT26-hHER2, or Q7D × 3 on days 12, 19, and 26 for JIMT-1). DS-8201a (T-DXd, 5.4 mg kg^−1^/10 mg kg^−1^, iv) was given once on day 7 for CT26-hHER2 or every 3 weeks (Q3W × 2; on days 12 and 33 for JIMT-1/SKOV3), depending on the study design.

Tumor growth and body weight were recorded every 2 to 4 days with a digital caliper until study termination (day 21 for CT26-hHER2 and day 44 for JIMT-1 models). Tumor volume was estimated using the equation: (length × width^2^)/2. At the conclusion of the experiment, tumors were harvested, weighed, and subsequently processed for histological and immunohistochemical examinations. For mechanistic analysis, paraffin sections were stained for H&E, Ki-67, γ-H2AX, and p-HER2 (Tyr^1221/1222^) to evaluate cell proliferation, DNA damage, and receptor activation.

### Histological and IF analysis

At the end of treatment, tumors and major organs were excised, fixed in 4% paraformaldehyde, and embedded in paraffin. Sections (4 μm) were prepared for histopathological evaluation using H&E staining. For IHC, deparaffinized tumor sections were incubated with anti-HER2 antibody (Cell Signaling Technology, no. 4290; 1:100) and anti-Ki-67 antibody (Abcam, ab15580; 1:200), a nuclear proliferation marker. For IF, sections were stained with antibodies against p-HER2 (Cell Signaling Technology, no. 2249; 1:100) and γ-H2AX (phospho-S139; Abcam, ab26350; 1:250), followed by Alexa Fluor–conjugated secondary antibodies (488/594; Invitrogen, A-11008; 1:500). Nuclei were counterstained with Hoechst 33342 (Invitrogen, H3570; 1 μg ml^−1^, 5 min). Fluorescence images were captured with a Nikon A1RS confocal microscope, and signal intensity was quantified using ImageJ software.

### Statistical analysis

Statistical analyses were performed using GraphPad Prism version 9.0 (GraphPad Software, USA). For flow cytometry, IF, IHC, ELISA, and tumor growth datasets, intergroup differences were analyzed by one-way or two-way analysis of variance (ANOVA) followed by Tukey’s post hoc multiple comparisons test. IC_50_ values were obtained through nonlinear regression using a four-parameter logistic model. Comparisons between two independent groups were assessed using an unpaired, two-tailed Student’s *t* test. Data are presented as means ± SD unless otherwise stated. Statistical significance was defined as follows: *P < 0.05; **P < 0.01; ***P < 0.001; ****P < 0.0001; n.s., no significant difference.
