# Supplementary material for: Albumin binding improves the pharmacokinetics and therapeutic efficacy of a 177Lu-labeled HER2 Fab radioconjugate
Source: Sci Adv. 2026 Jul 17;12(29):eaee4052. doi: 10.1126/sciadv.aee4052 (PMC13378577; doi:10.1126/sciadv.aee4052)
Supplement: Supplementary file 1 — Figs. S1 to S34 Tables S1 to S3 [file sciadv.aee4052_sm.pdf]

Science Advances
AAAS

# Supplementary Materials for

## Albumin binding improves the pharmacokinetics and therapeutic efficacy of a $^{177}$Lu-labeled HER2 Fab radioconjugate

Peifei Liu *et al.*

Corresponding author: Jingjing Zhang, j.zhang@nus.edu.sg; Xiaoyuan Chen, chen9647@gmail.com; Jinming Yu, sdyujinming@126.com; Bingyu Li, libingyu006@163.com; Jie Liu, linchuangliujie@163.com

*Sci. Adv.* **12**, eaee4052 (2026)
DOI: 10.1126/sciadv.aee4052

**This PDF file includes:**

Figs. S1 to S34
Tables S1 to S3

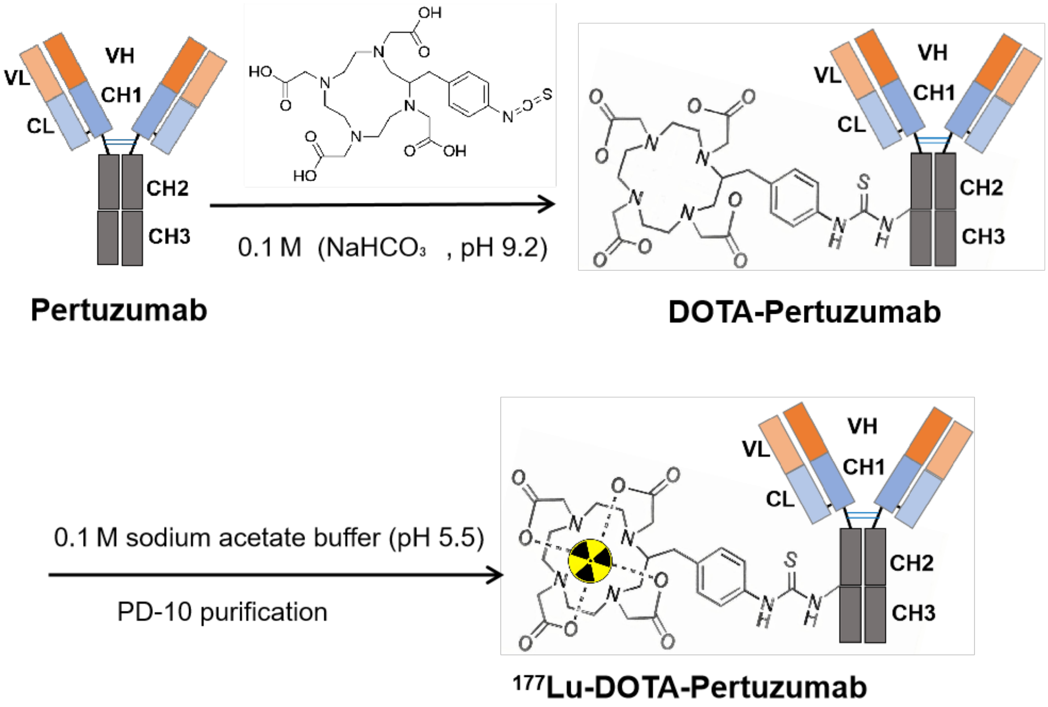


**Fig. S1. Schematic illustration of the synthesis of $^{177}$Lu-DOTA-pertuzumab.** Pertuzumab was conjugated to the bifunctional chelator p-SCN-Bn-DOTA (Macrocyclics, USA) via random coupling to lysine residues within the antibody, generating DOTA-pertuzumab. Subsequent coordination with the β$^{-}$-emitter $^{177}$Lu yielded $^{177}$Lu-DOTA-pertuzumab. The schematic depicts the antibody domains (VH, VL, CH1, CL, CH2, CH3) and the radiometal-chelator

complex.

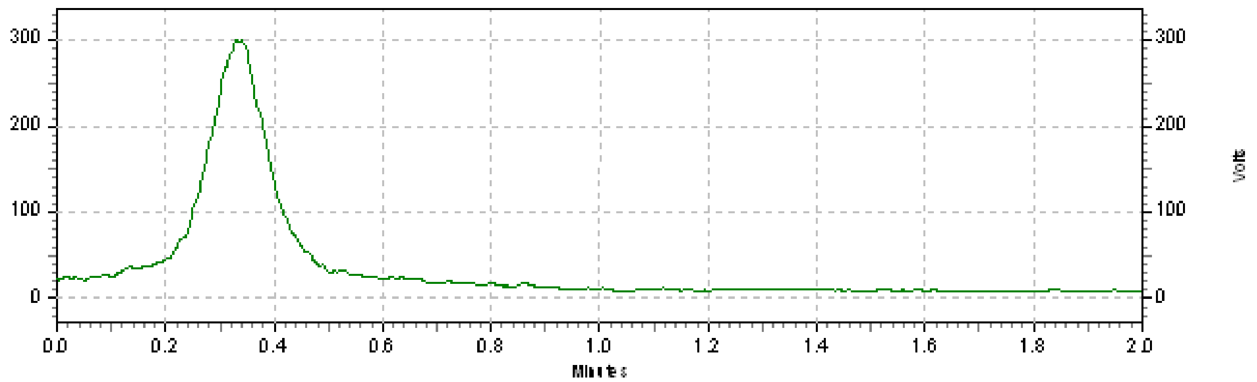


| Retention Time (min) | Peak Area | Area Percentage (%) | Peak Height | Percentage (%) |
|---|---|---|---|---|
| 0.338 | 2,071,838 | 99.82 | 270,772 | 99.168 |
| 1.117 | 3,744 | 0.18 | 2,271 | 0.832 |

**Fig. S2. Radio-thin-layer chromatography (radio-TLC) of $^{177}$Lu-DOTA-pertuzumab.** Representative chromatogram of purified $^{177}$Lu-DOTA-pertuzumab analyzed on silica gel ITLC-SG strips using 0.1 mol·L$^{-1}$ citrate buffer (pH 5.0) as the mobile phase. A 2 μL aliquot of the labeling solution (~0.37 MBq) was applied, and activity distribution was recorded using a radio-TLC scanner (ScanRAM, LabLogic, UK). The chromatogram shows a single predominant peak at $t_R$ = 0.338 min, accounting for 99.82% (area) / 99.168% (peak height), indicating a radiochemical purity of 99.8% (RCP > 95%). A minor peak at $t_R$ = 1.117 min represents 0.18% (area) / 0.832% (peak height). Peak statistics are summarized in the accompanying table.

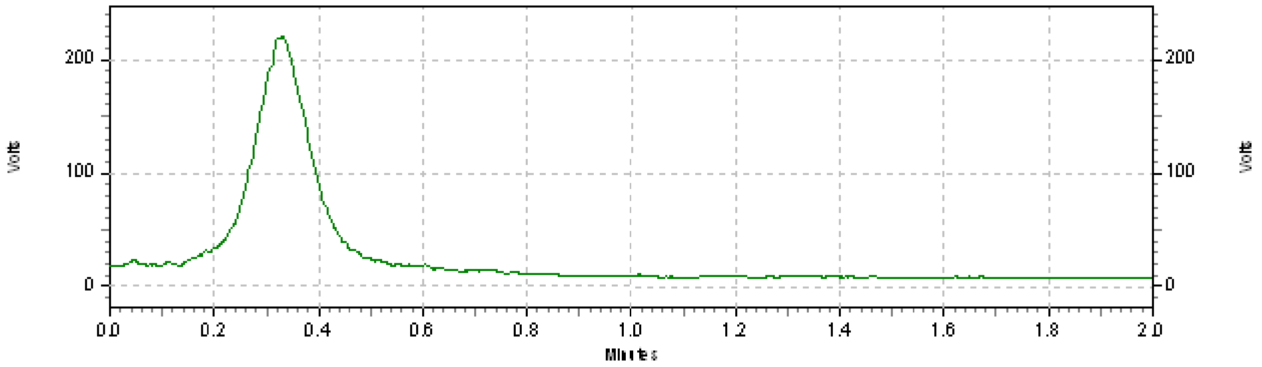


| Retention Time (min) | Peak Area | Area Percentage (%) | Peak Height | Percentage (%) |
|---|---|---|---|---|
| 0.332 | 1,530,714 | 99.65 | 202,402 | 99.118 |
| 1.157 | 5,381 | 0.35 | 1,802 | 0.882 |

**Fig. S3. Radio-thin-layer chromatography (radio-TLC) of $^{177}$Lu-DOTA-Fab-ABD.** Representative chroma togram of purified $^{177}$Lu-DOTA-Fab-ABD analyzed on silica gel ITLC-SG strips using 0.1 mol·L$^{-1}$ citrate buffer (pH 5.0) as the mobile phase. A 2 μL aliquot of the labeling mixture (~0.37 MBq) was applied, a nd radioactivity was measured using a radio-TLC scanner (ScanRAM, LabLogic, UK). The chromatogram shows a dominant peak at $t_R$ = 0.332 min corresponding to $^{177}$Lu-DOTA-Fab-ABD, with a radiochemical purity of 99.65% (area). A minor peak at $t_R$ = 1.157 min accounted for 0.35% (area). Peak statistics are summarized in the accompanying table.

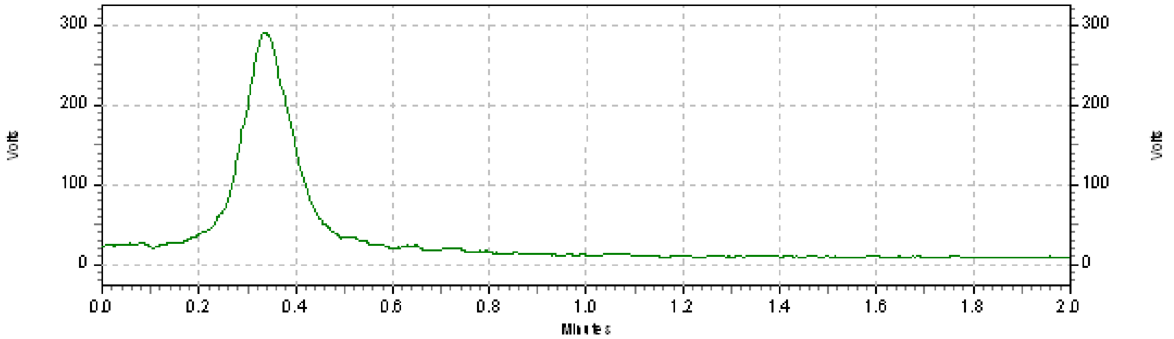


**Fig. S4. Radio-thin-layer chromatography (radio-TLC) of $^{177}$Lu-DOTA-Fab.** Representative chromatogram of purified $^{177}$Lu-DOTA-Fab analyzed on silica gel ITLC-SG strips using 0.1 mol·L$^{-1}$ citrate buffer (pH 5.0) as the

mobile phase. A 2 μL aliquot of the labeling mixture (~0.37 MBq) was applied, and radioactivity distribution was measured using a radio-TLC scanner (ScanRAM, LabLogic, UK).

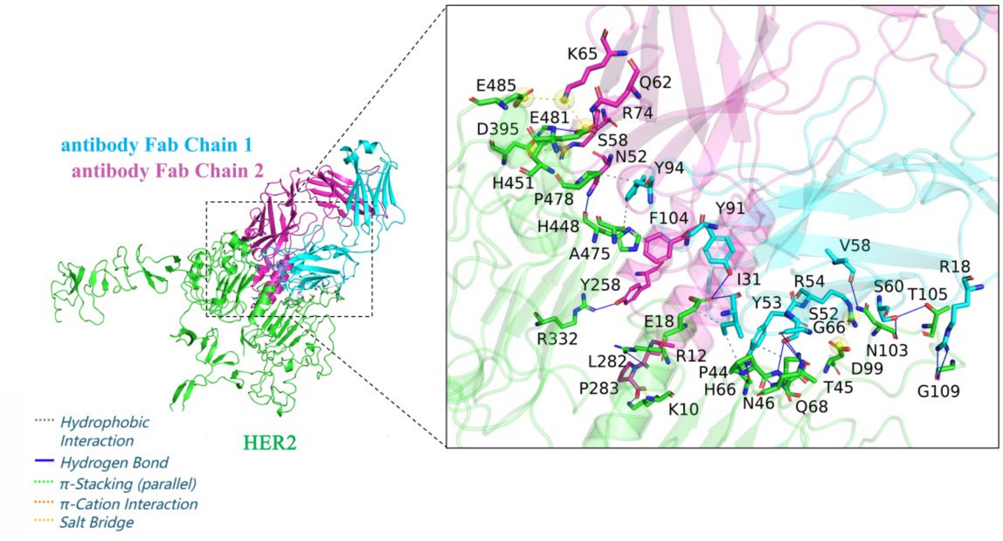


**Fig. S5. Structural model of the HER2 ectodomain (ECD) in complex with a HER2-specific Fab. Left,** Overall architecture of the complex. The HER2 ECD is shown in green (cartoon), and the Fab polypeptide chains are shown in cyan (chain 1) and magenta (chain 2). Right, enlarged view of the boxed region highlighting epitope-paratope interactions. Interaction types are annotated according to the legend: hydrophobic interactions (grey dotted lines), hydrogen bonds (blue lines), π-π stacking (green lines; none detected), π-cation interactions (orange dotted lines; none detected), and salt bridges (yellow dashed lines). Interface analysis identified 7 hydrophobic pairs, 20 hydrogen bonds, and 4 salt bridges, with no π-π or π-cation interactions. The calculated binding free energy ($\Delta G$ = -16.6 kcal·mol$^{-1}$) indicates a stable epitope-paratope interface.

The Fab was genetically fused with an albumin-binding domain (ABD). The albumin-binding domain (ABD) sequence used in this study is based on a previously reported albumin-binding domain *(44)*. The amino acid sequence is:

SLAEAKVLANRELDKYGVSDFYKRLINKAKTVEGVEALKLHILAALP.

| Model | Binding free energy ΔG (kcal·mol$^{-}$1) | Hydrophobic pairs (count) | Hydrogen bonds (count) | Salt bridges (count) | **π-π stacking (count)** | **π-cation interactions (count)** |
|---|---|---|---|---|---|---|
| Fab-ABD | -16.6 | 7 | 20 | 4 | 0 | 0 |
| Fab-ABD: Albumin | -12.1 | 3 | 8 | 1 | 0 | 0 |

**Table S1. Effect of albumin binding on the HER2 interaction of Fab-ABD.** Interface analysis showed that Fab-ABD alone exhibited a binding free energy of ΔG = -16.6 kcal·mol$^{-1}$ with the HER2 ectodomain, supported by 7 hydrophobic contacts, 20 hydrogen bonds, and 4 salt bridges. When Fab-ABD was pre-bound to albumin, its subsequent interaction with the HER2 ectodomain weakened (ΔG = -12.1 kcal·mol$^{-1}$), with reduced hydrophobic contacts (3), hydrogen bonds (8), and salt bridges (1). No π-π stacking or π-cation interactions were detected in either complex. These findings indicate that albumin engagement, while extending the circulating half-life of Fab-ABD, partially diminishes its intrinsic HER2 binding affinity.

Compared with **Figure S5**, the HER2 ECD binding in Fig. 2a shows weaker affinity (ΔG -12.1 vs -16.6 kcal·mol$^{-1}$), reflecting fewer hydrogen bonds and salt bridges. When Fab-ABD is pre-bound to albumin, the HER2 binding is partially attenuated, consistent with the reduced interaction counts (**Table S1**). These findings suggest that albumin binding extends half-life at the cost of modestly reduced HER2 affinity, which remains sufficient for effective targeting. Although albumin association slightly attenuated the apparent binding affinity to HER2, this modest reduction is likely offset by the prolonged circulation time and enhanced systemic exposure conferred by albumin binding, thereby supporting effective tumor accumulation *in vivo*.

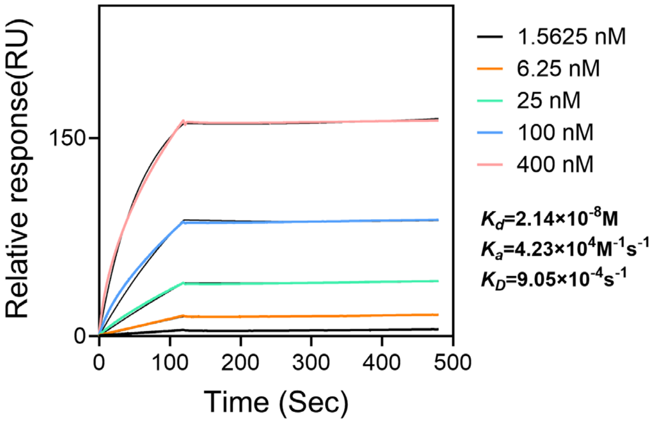


**Fig. S6. Surface plasmon resonance (SPR) sensorgrams of pertuzumab binding to the HER2 ECD.** Binding kinetics were evaluated by injecting serial dilutions of pertuzumab (1.5625, 6.25, 25, 100, and 400 nM) over immobilized HER2 ECD. Sensorgrams display concentration-dependent association and dissociation phases, with global fitting yielding kinetic constants of $K_d = 2.14 \times 10^{-8}$ M, $K_a = 4.23 \times 10^4$ $M^{-1}$. $s^{-1}$, and $K_D = 9.05 \times 10^{-4}$ $s^{-1}$. These parameters confirm high-affinity, nanomolar-range binding consistent with the established HER2 recognition profile of pertuzumab.

| **Model** | **Equilibrium dissociation constant $K_d$ (M)** | **Association rate constant $K_a$ ($M^{-1}\cdot s^{-1}$)** | **Dissociation rate constant $K_D$ ($s^{-1}$)** |
|---|---|---|---|
| Pertuzumab : HER2 | $2.14 \times 10^{-8}$ | $4.23 \times 10^{4}$ | $9.05 \times 10^{-4}$ |
| Fab-ABD : HER2 | $1.95 \times 10^{-8}$ | $5.36 \times 10^{4}$ | $1.04 \times 10^{-3}$ |
| Fab : HER2 | $1.99 \times 10^{-8}$ | $7.28 \times 10^{4}$ | $1.45 \times 10^{-3}$ |

**Table S2. SPR analysis of HER2 binding.** SPR sensorgrams show the binding kinetics of pertuzumab, Fab, and Fab-ABD to immobilized HER2 ectodomain. All constructs exhibited nanomolar affinity ($K_d \approx 2 \times 10^{-8}$ M). Fab and Fab-ABD displayed slightly faster association and dissociation rates compared with pertuzumab, but remained within the same kinetic order of magnitude, indicating preserved high-affinity HER2 recognition.

Overall, the equilibrium affinity of Fab, Fab-ABD, and full-length pertuzumab toward HER2 is

comparable, all in the nanomolar range. The Fab variant bound and dissociated somewhat faster, whereas ABD fusion slightly slowed both processes, but the differences are minor and do not alter the overall binding level.

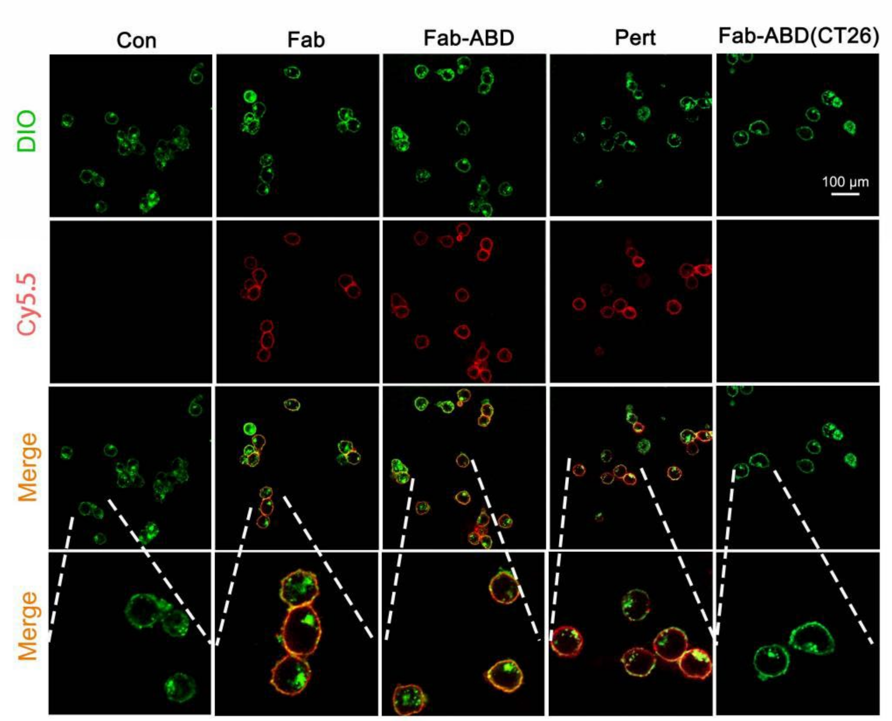


**Fig. S7. Confocal microscopy of HER2 binding in CT26-hHER2 cells and hHER2-negative CT26 controls.** CT26-hHER2 and parental CT26 cells were stained with the membrane dye DiO (green) and incubated with Cy5.5-labeled antibody constructs, including Fab, Fab-ABD, and pertuzumab (Pert). "Con" denotes untreated control cells, whereas "Fab-ABD (CT26)" represents hHER2-negative CT26 cells incubated with Fab-ABD as a negative control. Strong Cy5.5 signals were observed on hHER2-positive CT26-hHER2 cells for all three constructs, whereas no antibody binding was detected in hHER2-negative CT26 cells. Merged images show co-localization of membrane (green) and antibody (red) signals, confirming HER2-specific binding. These fluorescence images originate from the same confocal dataset shown in Fig. 2F but are displayed without the DIC channel to improve visualization of the fluorescence signals. Scale bar, 100 µm.

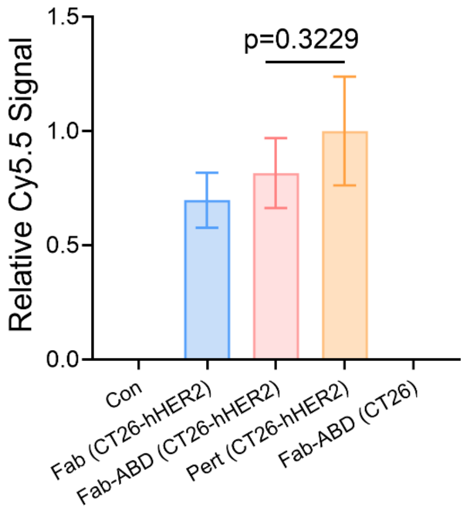


**Fig. S8. Quantitative analysis of HER2 binding by Fab, Fab-ABD, and pertuzumab.** Relative Cy5.5 fluorescence was measured in CT26-hHER2 cells incubated with Fab, Fab-ABD, or pertuzumab (Pert), with untreated CT26-hHER2 cells serving as controls and hHER2-negative CT26 cells included as a negative reference. All three constructs showed significantly higher fluorescence intensity than control cells, confirming HER2-specific binding. Fab-ABD displayed slightly lower binding intensity than pertuzumab, although the difference was not statistically significant (P = 0.3229). Data are presented as mean ± SEM (n = 3).

These results demonstrate that Fab, Fab-ABD, and pertuzumab all effectively recognize hHER2-positive cells at the plasma membrane level. The ABD fusion preserves HER2 binding, with only minor, non-significant differences compared to the full-length antibody.

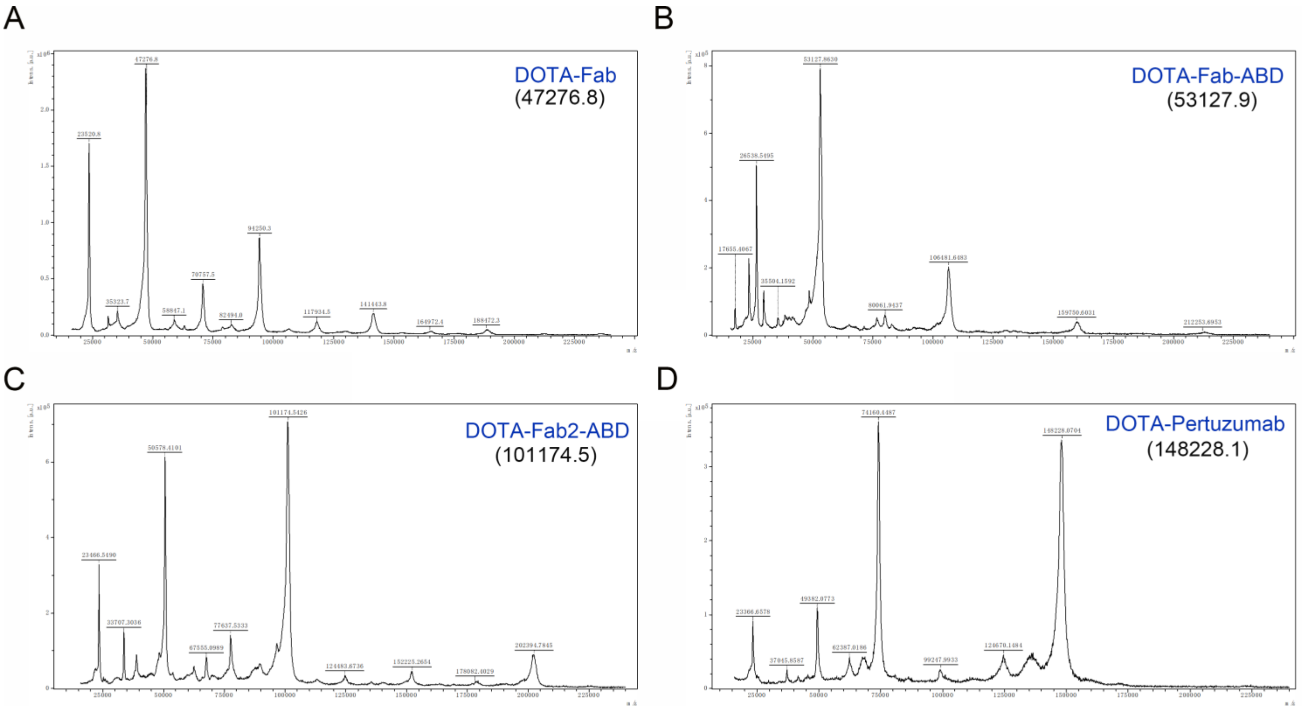


**Fig. S9. Mass spectrometric characterization of DOTA-conjugated antibody constructs.** (**A**) Deconvoluted mass spectrum of DOTA-Fab showing a predominant molecular weight of 47276.8 Da. (**B**) Deconvoluted mass spectrum of DOTA-Fab-ABD with a major peak at 53127.9 Da, consistent with successful fusion of the albumin-binding domain (ABD). (**C**) Deconvoluted mass spectrum of DOTA-Fab2-ABD demonstrating a principal molecular weight of 101174.5 Da, corresponding to the dimeric Fab format fused with ABD. (**D**) Deconvoluted mass spectrum of DOTA-pertuzumab with a dominant peak at 148228.1 Da.

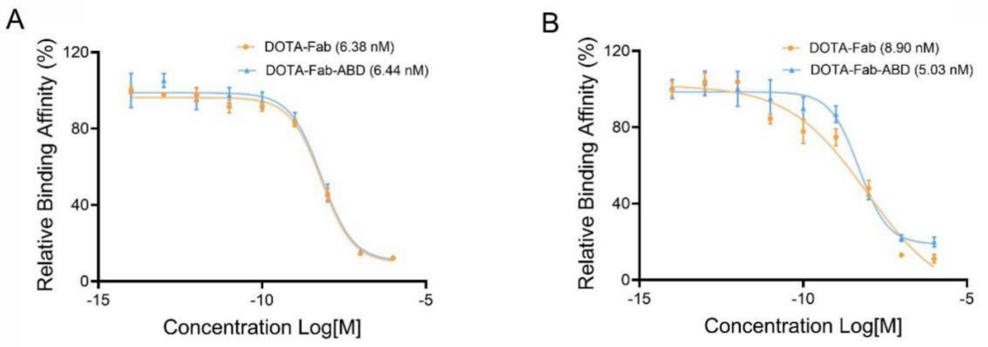

**Fig. S10. Competitive binding assay of DOTA-Fab and DOTA-Fab-ABD in HER2-positive cells.** (**A**) Competitive binding curves of DOTA-Fab and DOTA-Fab-ABD on SKOV3 cells, yielding $IC_{50}$ values of 6.38 nM for DOTA-Fab and 6.44 nM for DOTA-Fab-ABD. (**B**) Competitive binding in CT26-hHER2 cells, with $IC_{50}$ values of 8.90 nM for DOTA-Fab and 5.03 nM for DOTA-Fab-ABD. Data represent mean ± SEM (n = 3–4).

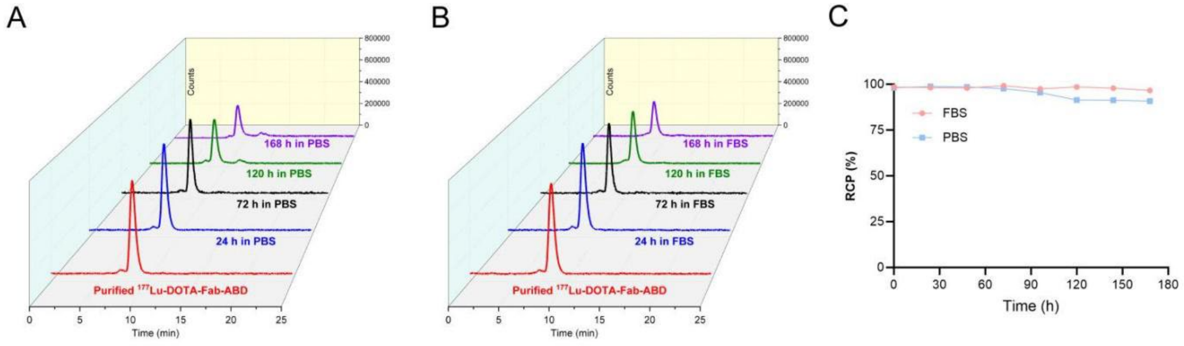


**Fig. S11. *In vitro* stability analysis of $^{177}$Lu-DOTA-Fab-ABD in PBS and FBS.** (**A**) Radio-HPLC chromatograms of purified $^{177}$Lu-DOTA-Fab-ABD and samples incubated in PBS at 37 °C for 24, 72, 120, and 168 h. The radioconjugate maintained a dominant intact peak over time, indicating high stability in buffer conditions. (**B**) Radio-HPLC chromatograms of $^{177}$Lu-DOTA-Fab-ABD incubated in fetal bovine serum (FBS) at 37 °C for 24, 72, 120, and 168 h. The major radiolabeled peak remained largely unchanged, demonstrating good serum stability with minimal degradation. (**C**) Quantitative analysis of radiochemical purity (RCP) over time in PBS and FBS. $^{177}$Lu-DOTA-Fab-ABD retained high radiochemical purity (>90%) throughout the 168 h incubation period in both media, confirming its favorable *in vitro* stability profile. Data are presented as mean ± SD where applicable.

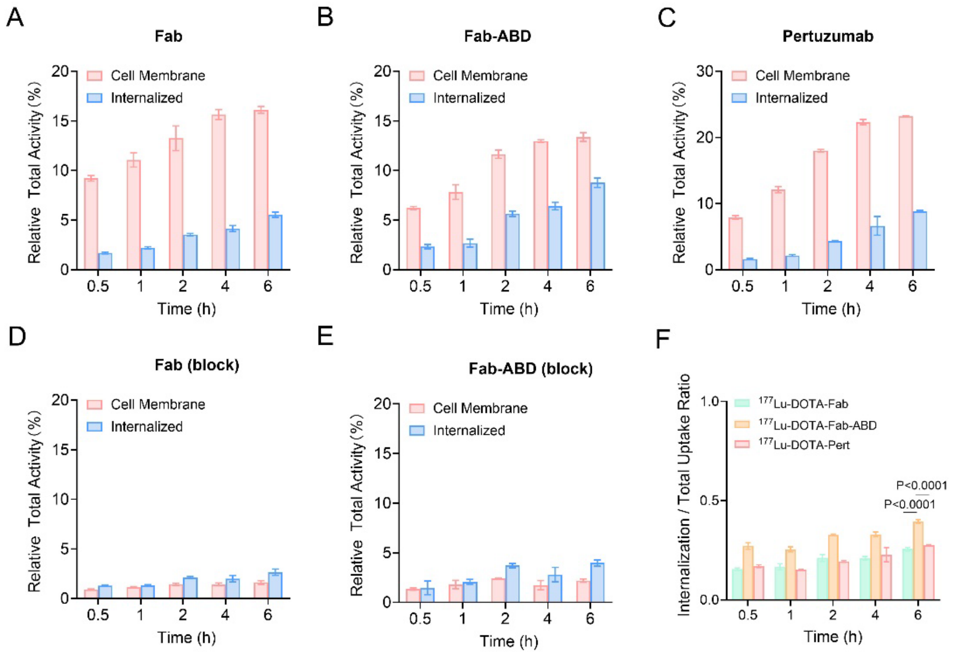


**Fig. S12. Cellular uptake and internalization of $^{177}$Lu-labeled Fab, Fab-ABD, and pertuzumab in SKOV3 cells.** Time-dependent cellular uptake and internalization of $^{177}$Lu-DOTA-Fab (**A**), $^{177}$Lu-DOTA-Fab-ABD (**B**), and $^{177}$Lu-DOTA-pertuzumab (**C**). (**D, E**) Blocking studies using excess unlabeled antibody confirmed HER2-specific binding. (**F**) Time-dependent internalization-to-total uptake ratios of the three radioconjugates over 6 h. At 6 h post-incubation, Fab-ABD achieved an internalized fraction of 8.78 ± 0.41%, comparable to that of pertuzumab (8.85 ± 0.10%), although its total cellular uptake was slightly lower. As a result, Fab-ABD displayed a significantly higher internalization-to-total uptake ratio (0.40 ± 0.01) compared with pertuzumab (0.28 ± 0.00, $P < 0.0001$). Data are presented as mean ± SEM (n = 4).

These results demonstrate that Fab-ABD achieves internalization levels equivalent to full-length pertuzumab, while exhibiting a higher internalization efficiency relative to total uptake. This suggests that ABD fusion preserves effective HER2-mediated endocytosis despite lower overall uptake.

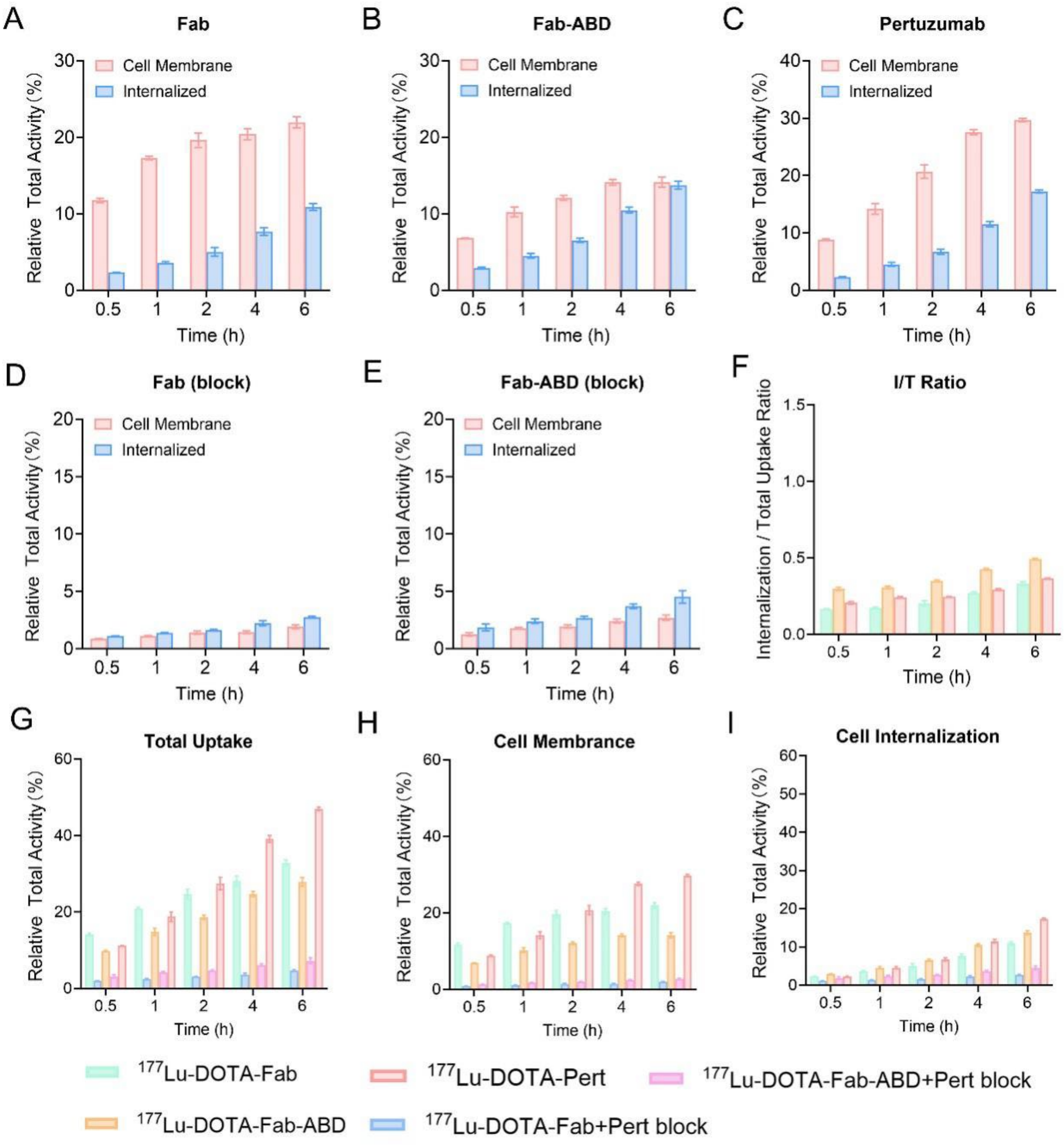


**Fig. S13. Cellular uptake and internalization of $^{177}$Lu-labeled antibodies in CT26-hHER2 cells.** (**A–C**) Time-dependent uptake and internalization of $^{177}$Lu-DOTA-Fab (**A**), $^{177}$Lu-DOTA-Fab-ABD (**B**), and $^{177}$Lu-DOTA-pertuzumab (**C**) in CT26-hHER2 cells. (**D, E**) Blocking studies using excess unlabeled pertuzumab confirmed HER2-specific binding. (**F**) Internalization-to-total uptake (I/T) ratios for Fab, Fab-ABD, and pertuzumab. Comparison of total uptake (**G**), membrane binding (**H**), and internalization (**I**) across the three constructs, with corresponding blocking controls. Data are presented as mean ± SEM (n = 4).

The uptake and internalization patterns of Fab-ABD in CT26-hHER2 cells were highly consistent with

those observed in SKOV3 cells. At 6 h, total uptake was 32.85 ± 0.64% for Fab and 27.84 ± 1.00% for Fab-ABD, both effectively blocked by pertuzumab (4.65 ± 0.16% and 7.21 ± 0.65%, respectively). The internalization ratio again favored Fab-ABD (49.30 ± 0.49%) over pertuzumab (36.71 ± 0.21%), demonstrating that Fab-ABD maintains robust and reproducible HER2-specific binding and internalization across different HER2-positive tumor cell models.

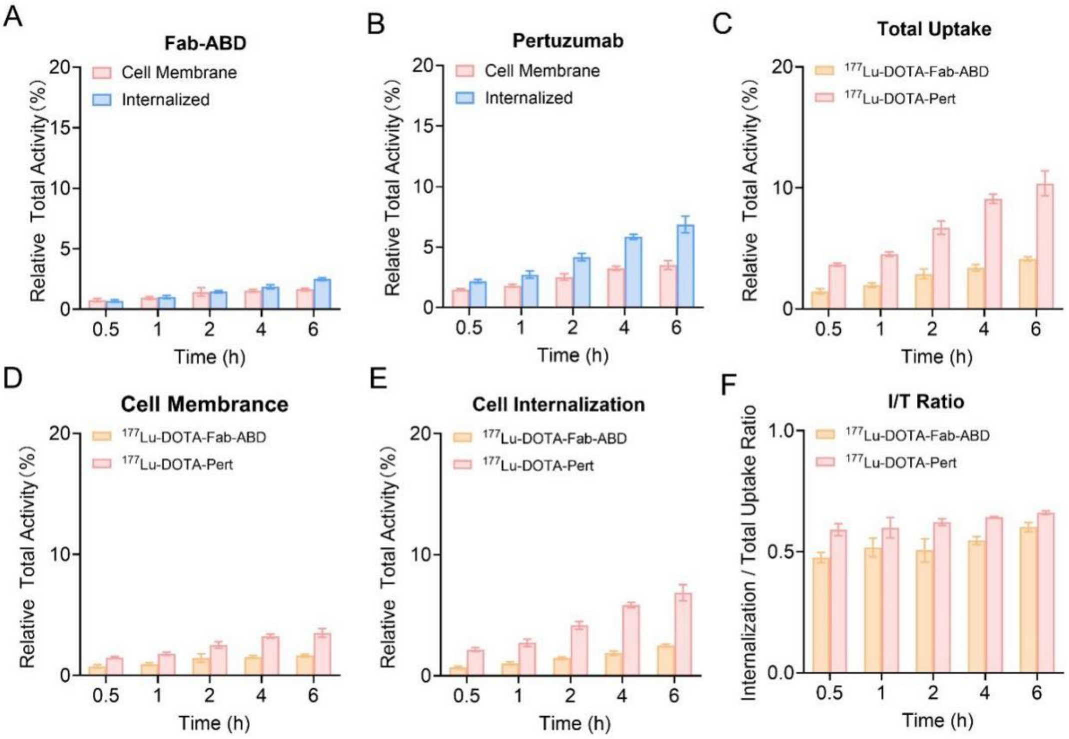


**Fig. S14. Cellular uptake and internalization of $^{177}$Lu-labeled Fab-ABD and pertuzumab in HER2-negative CT26 cells.** Time-dependent uptake and internalization of $^{177}$Lu-DOTA-Fab-ABD (A) and $^{177}$Lu-DOTA-pertuzumab (B) in parental CT26 cells lacking human HER2 expression. Comparative analysis of total uptake (**C**), membrane association (**D**), internalization (**E**), and internalization-to-total uptake ratios (**F**). Both Fab-ABD and pertuzumab showed low overall uptake, consistent with the absence of HER2. Pertuzumab exhibited relatively higher non-specific uptake than Fab-ABD. Data are presented as mean ± SEM (n = 4).

These findings confirm that in HER2-negative cells, Fab-ABD shows minimal non-specific uptake, whereas full-length pertuzumab exhibits higher off-target binding. Such non-specific accumulation is independent of Fab binding sites and may contribute to unwanted uptake in non-target tissues in vivo, potentially increasing radiation-associated toxicity.

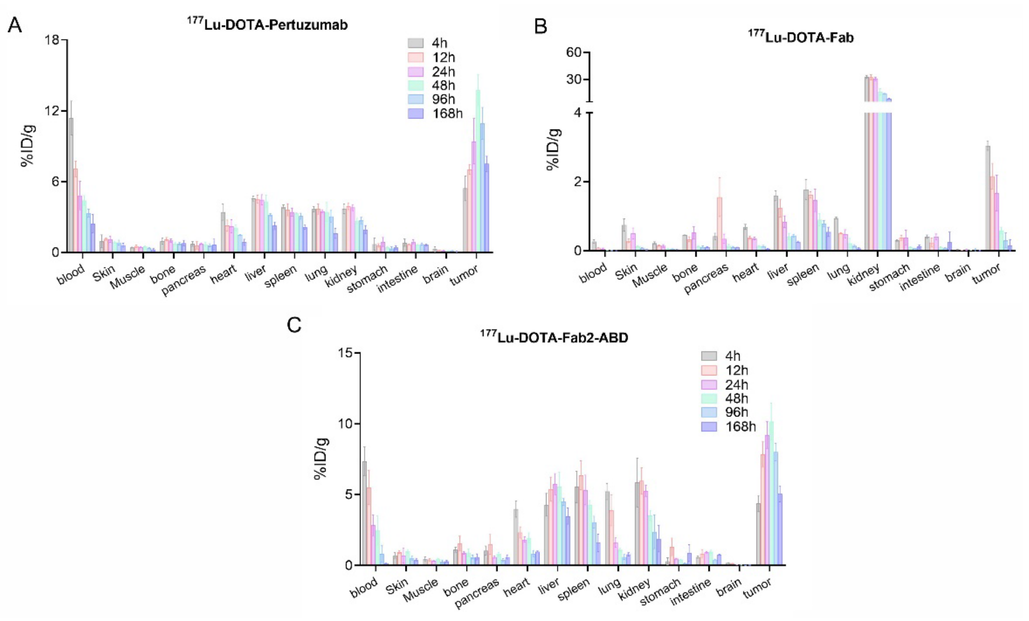


**Fig. S15. Biodistribution of $^{177}$Lu-labeled antibodies in CT26-hHER2 tumor-bearing mice.** (**A**) Biodistribution of $^{177}$Lu-DOTA-pertuzumab at 4, 12, 24, 48, 96, and 168 h post-injection. (**B**) Biodistribution of $^{177}$Lu-DOTA-Fab at the same time points. (**C**) Biodistribution of $^{177}$Lu-DOTA-Fab2-ABD. Data are presented as %ID/g (mean ± SEM, n = 3 per group).

These results highlight the pharmacokinetic contrast between full-length IgG and Fab fragments: $^{177}$Lu-DOTA-pertuzumab displays sustained blood circulation and tumor uptake, while $^{177}$Lu-DOTA-Fab demonstrates faster clearance and higher renal accumulation, reflecting its smaller molecular size and rapid renal filtration.

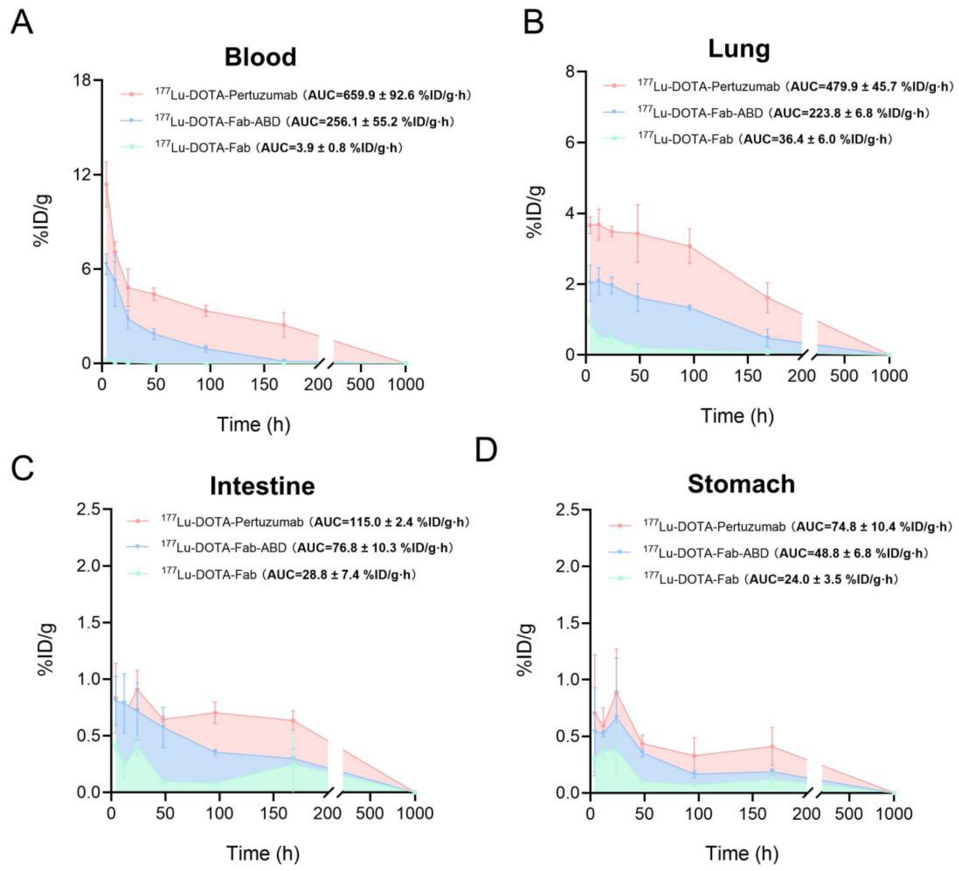


**Fig. S16. Pharmacokinetic biodistribution of $^{177}$Lu-labeled pertuzumab, Fab-ABD, and Fab in non-tumor tissues.** (**A**) Blood: time-activity curves of $^{177}$Lu-DOTA-pertuzumab, $^{177}$Lu-DOTA-Fab-ABD, and $^{177}$Lu-DOTA-Fab were generated. The area under the curve (AUC) values (mean ± SEM, n = 3) were 659.9 ± 92.6 for pertuzumab, 256.1 ± 55.2 for Fab-ABD, and 3.9 ± 0.8 for Fab. (**B**) Lung: time-activity curves were generated as described above. The AUC values (mean ± SEM, n = 3) were 479.9 ± 45.7 for pertuzumab, 223.8 ± 6.8 for Fab-ABD, and 36.4 ± 6.0 for Fab. (**C**) Intestine: time-activity curves were generated as described above. The AUC values (mean ± SEM, n = 3) were 115.0 ± 2.4 for pertuzumab, 76.8 ± 10.3 for Fab-ABD, and 28.8 ± 7.4 for Fab. (**D**) Stomach: time-activity curves were generated as described above. The AUC values (mean ± SEM, n = 3) were 74.8 ± 10.4 for pertuzumab, 48.8 ± 6.8 for Fab-ABD, and 24.0 ± 3.5 for Fab.

Compared with full-length pertuzumab, Fab and Fab-ABD showed markedly lower exposure (AUC) in blood and major normal organs, indicating reduced off-target radiation dose. The ABD fusion extended Fab circulation relative to unfused Fab, but maintained lower systemic retention than pertuzumab.

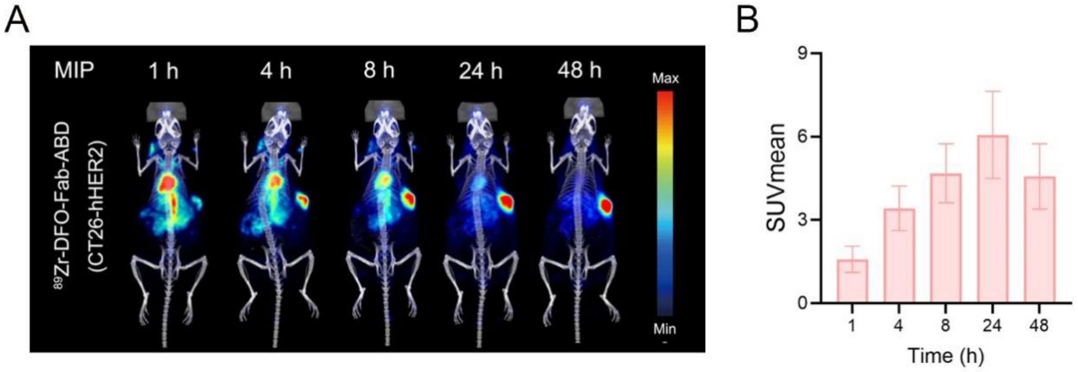


**Fig. S17. PET imaging of $^{89}$Zr-DFO-Fab-ABD in CT26-hHER2 tumor-bearing mice.** (**A**) Maximum-intensity projection (MIP) PET images obtained at 1, 4, 8, 24, and 48 h after injection of $^{89}$Zr-DFO-Fab-ABD show rapid and specific tumor accumulation with progressive clearance from non-tumor tissues, resulting in high tumor-to-background contrast at later time points. (**B**) Quantitative analysis of tumor uptake expressed as SUVmean at corresponding time points. Tumor uptake increased over time and reached a peak value of 6.06 ± 1.28 at 24 h post-injection, followed by a slight decline at 48 h while maintaining substantial retention. Data are presented as mean ± SD.

The PET signal distribution shown in Fig. S17 closely parallels that observed with $^{177}$Lu-DOTA-Fab-ABD SPECT imaging, confirming consistent in vivo targeting and pharmacokinetic behavior across

imaging modalities. The strong, persistent tumor visualization and low background uptake highlight the translational potential of Fab-ABD-based tracers for clinical HER2-targeted PET imaging.

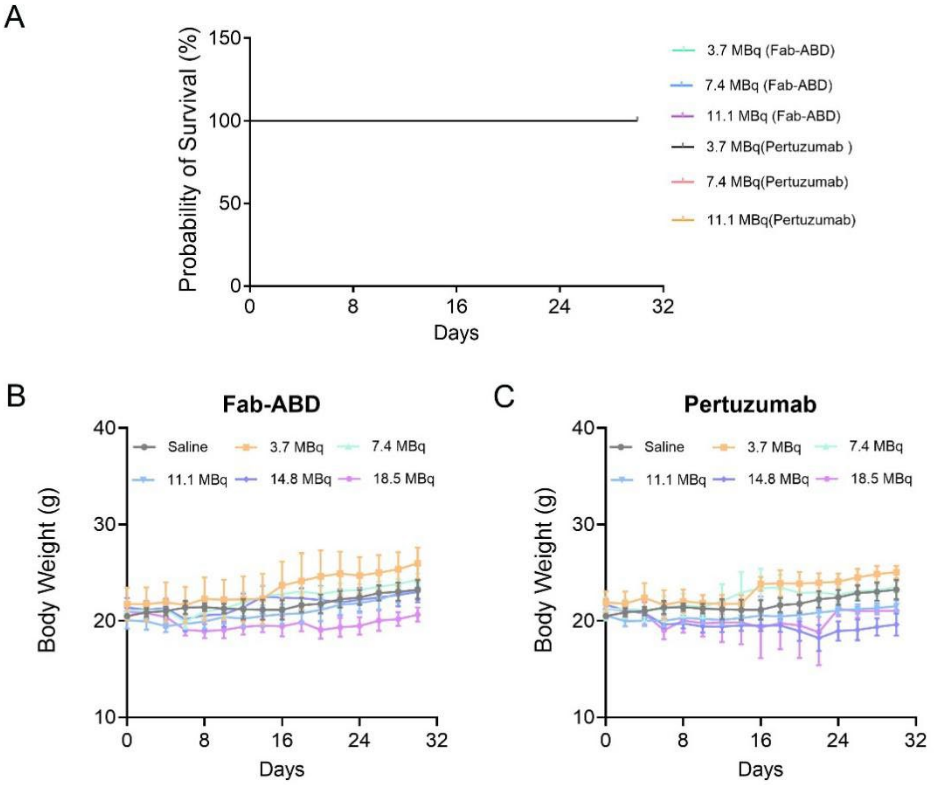


**Fig. S18. Safety evaluation of $^{177}$Lu-DOTA-Fab-ABD and $^{177}$Lu-DOTA-pertuzumab in healthy BALB/c mice.** (**A**) Kaplan-Meier survival analysis of mice treated with escalating doses of $^{177}$Lu-DOTA-Fab-ABD or $^{177}$Lu-DOTA-pertuzumab (3.7, 7.4, 11.1 MBq) showed no treatment-related mortality within 30 days. Body-weight monitoring across extended dose ranges (3.7–18.5 MBq) demonstrated dose-dependent but overall stable weight profiles for both $^{177}$Lu-DOTA-Fab-ABD(**B**) and $^{177}$Lu-DOTA-pertuzumab(**C**). Data represent mean ± SEM (n = 6).

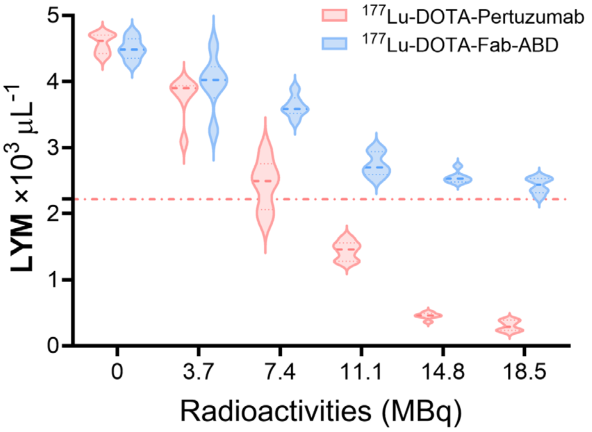


**Fig. S19. Hematological toxicity of $^{177}$Lu-DOTA-pertuzumab and $^{177}$Lu-DOTA-Fab-ABD.** Lymphocyte (LYM) counts in mice treated with escalating doses of $^{177}$Lu-DOTA-pertuzumab (red) or $^{177}$Lu-DOTA-Fab-ABD (blue) are presented as violin plots across injected activities ranging from 0 to 18.5 MBq. Red dashed lines denote the lower limit of the normal physiological range for BALB/c mice and serve as a reference for evaluating hematologic toxicity. $^{177}$Lu-DOTA-pertuzumab induced a pronounced dose-dependent decrease in lymphocyte levels, whereas $^{177}$Lu-DOTA-Fab-ABD maintained substantially higher counts under the same conditions. Data represent mean ± SEM (n = 6).

These findings suggest that Fab-ABD exhibits a more favorable hematological safety profile than full-length pertuzumab, with reduced bone marrow suppression at equivalent radioactivities.

| Parameter | Unit | Mean ± 95% CI (Range) | Reference interval |
| --- | --- | --- | --- |
| **RBCs** (Red Blood Cells) | $\times 10^6$ / μL | 10.09 (6.93 – 12.24)S | 6.93 – 12.24 |
| **WBCs** (White Blood Cells) | $\times 10^3$ / μL | 8.62 (3.48 – 14.03) | 3.48 – 14.03 |
| **HGB** (Hemoglobin) | g / dL | 15.6 (12.6 – 20.5) | 12.6 – 20.5 |
| **PLTs** (Platelets) | $\times 10^3$ / μL | 1007 (420 – 1698) | 420 – 1698 |
| **LYMs** (Lymphocytes) | $\times 10^3$ / μL | 5.90 (2.22 – 9.83) | 2.22 – 9.83 |

**Table S3. Hematological reference intervals for BALB/cAnNCrl mice (8–10 weeks, non-fasted).** Reference values represent the mean and observed physiological ranges for healthy BALB/cAnNCrl mice housed under standard conditions. Measurements were obtained using a HemaVet automated hematology analyzer and serve as baseline reference intervals for interpreting treatment-related hematologic changes. Source: Charles River Laboratories, *BALB/cAnNCrl Mouse Clinical Pathology Data (Hematology and Clinical Chemistry Reference Ranges)*, Wilmington, MA; 2008-2012.

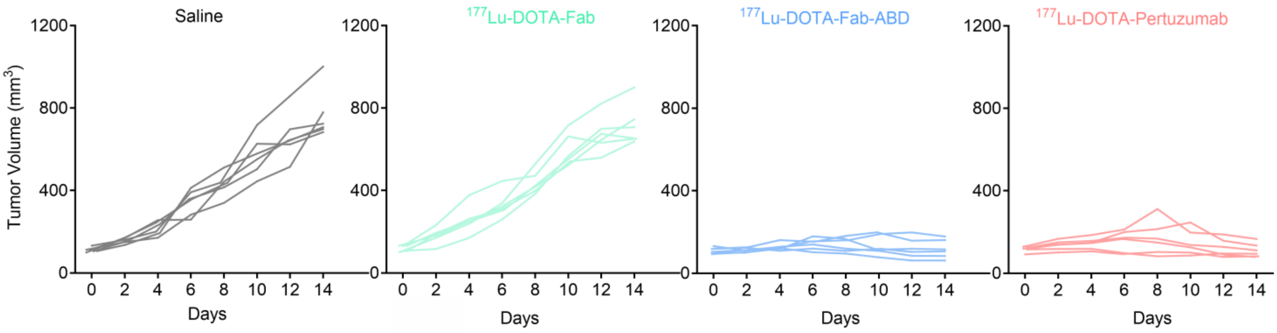


**Fig. S20. Tumor growth inhibition by $^{177}$Lu-labeled constructs in HER2-positive xenografts.** Individual tumor growth curves of mice treated with saline, $^{177}$Lu-DOTA-Fab, $^{177}$Lu-DOTA-Fab-ABD, or $^{177}$Lu-DOTA-pertuzumab (n = 6 per group). Tumors in the saline and Fab groups exhibited rapid progression, whereas both $^{177}$Lu-DOTA-Fab-ABD and $^{177}$Lu-DOTA-pertuzumab produced marked suppression of tumor growth over the 14-day observation period, demonstrating robust therapeutic efficacy in HER2-positive xenografts.

These results demonstrate that Fab-ABD achieves anti-tumor efficacy comparable to full-length pertuzumab, while unfused Fab shows limited therapeutic effect, underscoring the importance of ABD-mediated half-life extension in enabling effective radionuclide therapy.

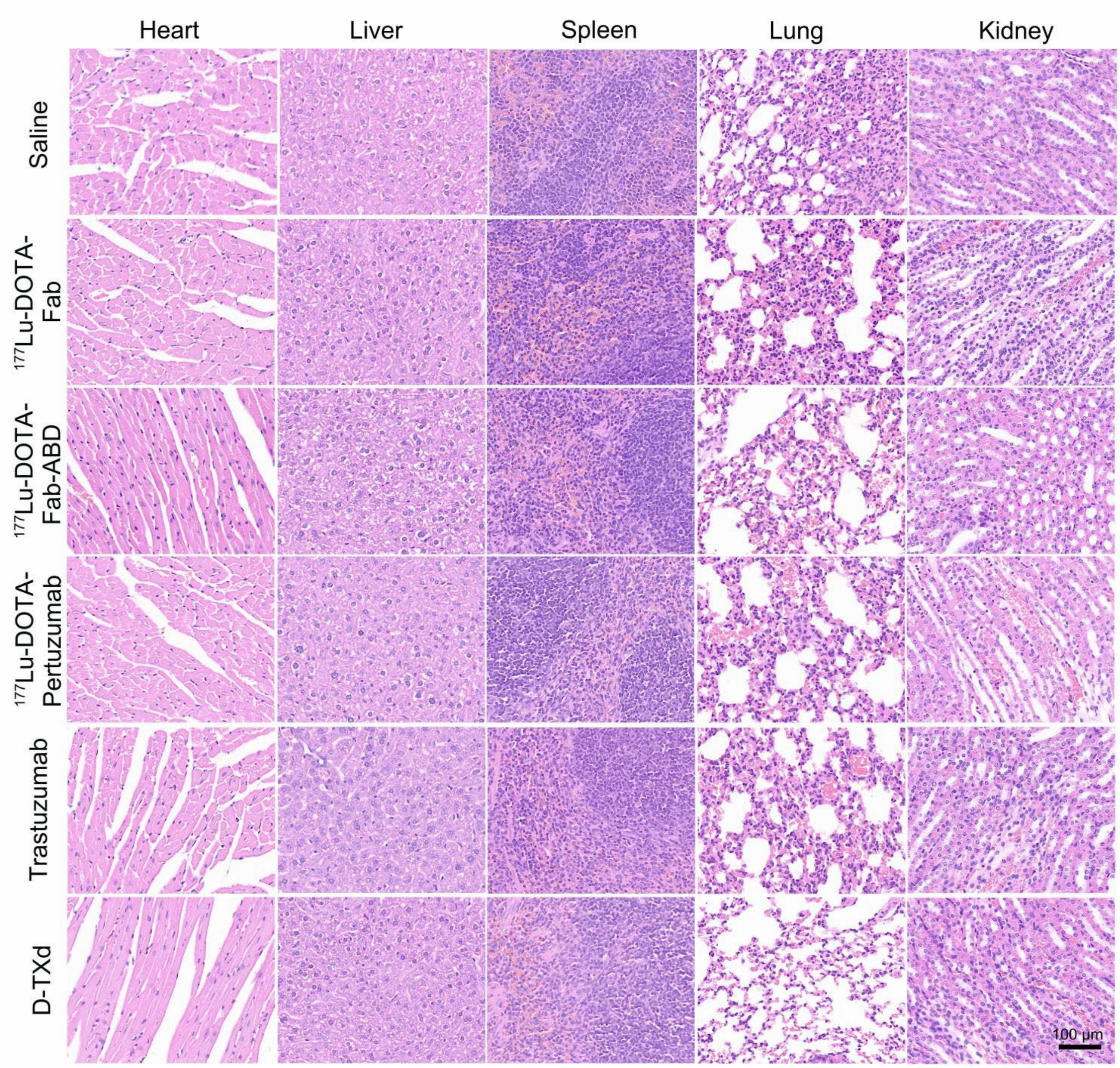


**Fig. S21. Histopathological evaluation of major organs after treatment with $^{177}$Lu-labeled constructs and controls.** H&E-stained sections of the heart, liver, spleen, lung, and kidney from mice treated with saline, $^{177}$Lu-DOTA-Fab, $^{177}$Lu-DOTA-Fab-ABD, $^{177}$Lu-DOTA-pertuzumab, trastuzumab, or T-DXd (scale bars, 100 μm). No treatment-related histopathological abnormalities were observed in any vital organ following administration of the $^{177}$Lu-labeled antibodies, with tissue morphology comparable to saline controls.

These results indicate that Fab-ABD and pertuzumab radio-conjugates are well tolerated in vivo, with no obvious damage to critical organs, further supporting their safety profile relative to standard HER2-targeted therapies such as trastuzumab and T-DXd.

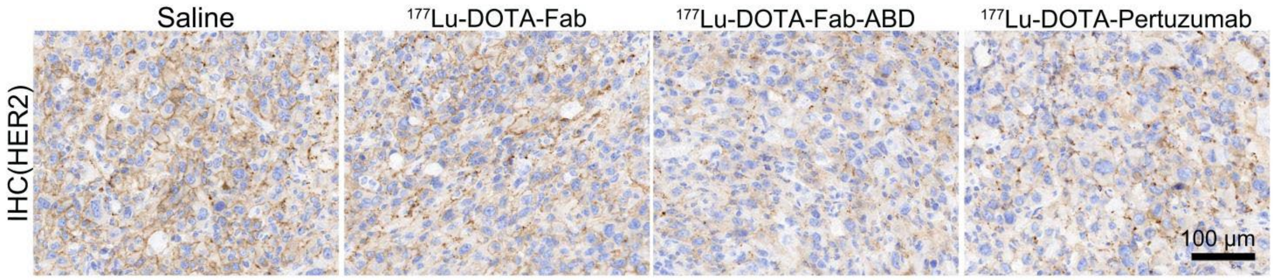


**Fig. S22. Immunohistochemical analysis of HER2 expression in tumors.** IHC staining of tumor sections from mice treated with saline, $^{177}$Lu-DOTA-Fab, $^{177}$Lu-DOTA-Fab-ABD, or $^{177}$Lu-DOTA-pertuzumab demonstrated uniformly strong HER2 expression across all treatment groups, indicating preserved target availability throughout the study. Scale bar, 100 µm.

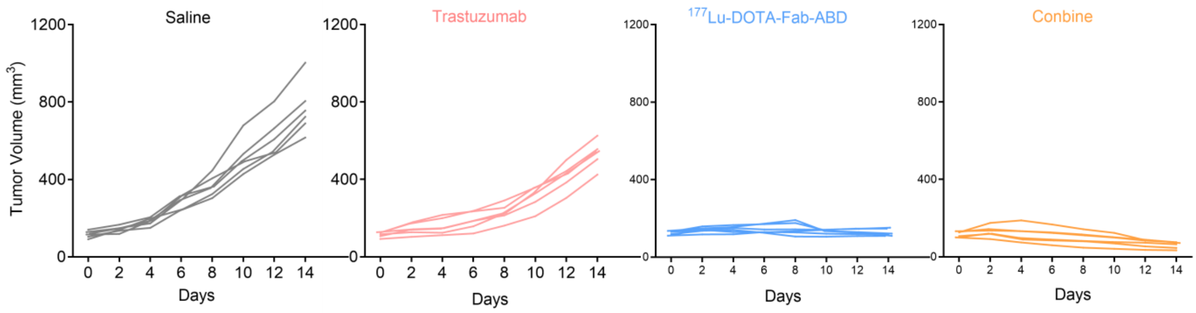


**Fig. S23. Antitumor efficacy of $^{177}$Lu-DOTA-Fab-ABD in combination with trastuzumab.** Individual tumor growth curves of mice treated with saline, trastuzumab, $^{177}$Lu-DOTA-Fab-ABD, or the combination of $^{177}$Lu-DOTA-Fab-ABD plus trastuzumab (n = 6 per group). Trastuzumab monotherapy produced partial tumor growth delay, whereas $^{177}$Lu-DOTA-Fab-ABD alone resulted in pronounced tumor suppression. The combination regimen further

enhanced therapeutic efficacy, with tumors exhibiting sustained regression throughout the 14-day evaluation period.

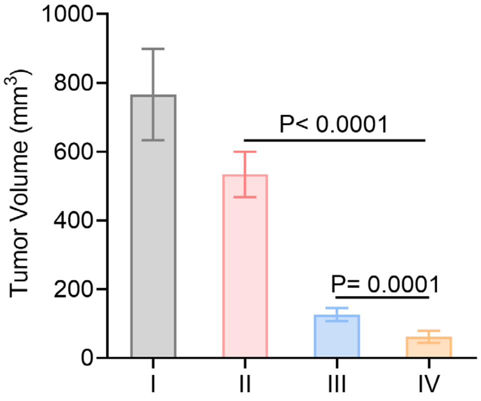


**Fig. S24. Quantitative analysis of tumor inhibition by trastuzumab, $^{177}$Lu-DOTA-Fab-ABD, and combination therapy.** Tumor volumes at day 14 post-treatment are shown for four groups: saline control, trastuzumab, $^{177}$Lu-DOTA-Fab-ABD, and the combination of $^{177}$Lu-DOTA-Fab-ABD plus trastuzumab. $^{177}$Lu-DOTA-Fab-ABD significantly suppressed tumor growth compared with both saline and trastuzumab monotherapy ($P = 0.0001$), and the combination regimen produced even greater tumor regression than either single agent ($P < 0.0001$). Data represent mean ± SEM ($n = 6$).

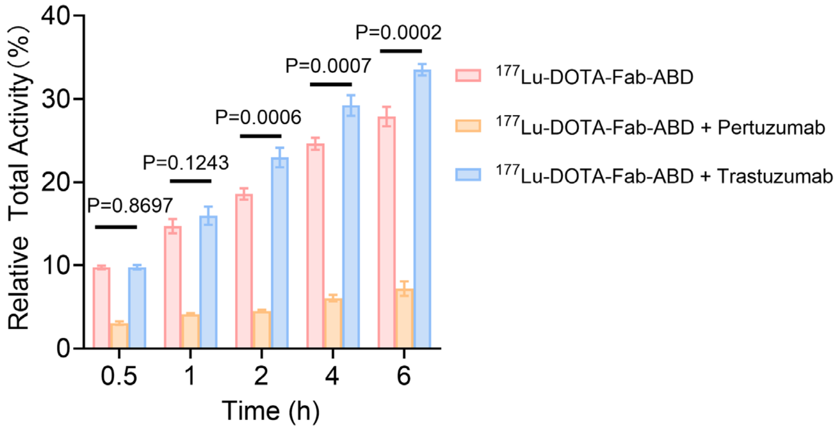

**Fig. S25. Effects of pertuzumab and trastuzumab preincubation on cellular uptake of $^{177}$Lu-DOTA-Fab-ABD.** Cellular uptake of $^{177}$Lu-DOTA-Fab-ABD was quantified in HER2-positive cells following co-incubation with unlabeled pertuzumab or trastuzumab. Preincubation with pertuzumab significantly reduced Fab-ABD uptake, indicating competitive blockade at overlapping HER2 epitopes. In contrast, trastuzumab preincubation enhanced Fab-ABD uptake, consistent with binding to distinct HER2 domains and a potential facilitation of receptor clustering or endocytosis. At 6 h, $^{177}$Lu-DOTA-Fab-ABD uptake was markedly increased in the trastuzumab co-incubation group compared with Fab-ABD alone ($P = 0.0002$). Data represent mean ± SEM ($n = 4$).

These results in Fig. S25 demonstrate that while pertuzumab competes with Fab-ABD for HER2 binding, trastuzumab acts synergistically to enhance Fab-ABD uptake, suggesting that combination therapy may increase tumor irradiation through complementary epitope engagement.

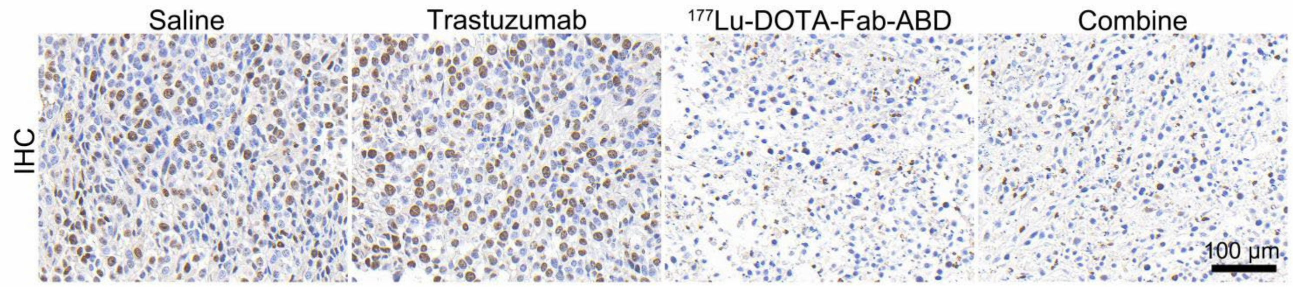


**Fig. S26. Immunohistochemistry of tumor sections after treatment.** Representative IHC images of tumors from mice treated with saline, trastuzumab, $^{177}$Lu-DOTA-Fab-ABD, or the combination of $^{177}$Lu-DOTA-Fab-ABD plus trastuzumab. Ki-67 staining revealed strong nuclear positivity in the saline and trastuzumab groups, whereas $^{177}$Lu-DOTA-Fab-ABD markedly reduced proliferative activity. The combination group exhibited the lowest Ki-67 signal, consistent with enhanced antiproliferative effects. Scale bar, 100 μm.

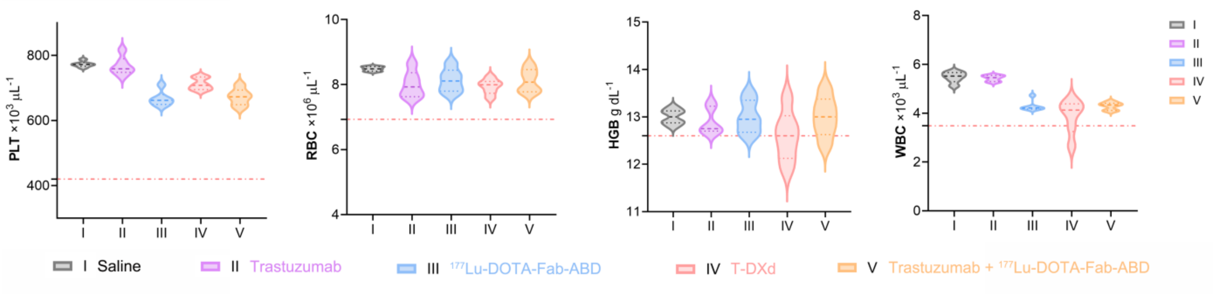


**Fig. S27. Hematological assessment after treatment with trastuzumab, $^{177}$Lu-DOTA-Fab-ABD, T-DXd, or combination therapy.** Violin plots show peripheral blood parameters: including platelet counts (PLT), red blood cell counts (RBC), hemoglobin levels (HGB), and white blood cell counts (WBC) in healthy BALB/c mice treated with saline, trastuzumab, $^{177}$Lu-DOTA-Fab-ABD, T-DXd, or the combination of trastuzumab plus $^{177}$Lu-DOTA-Fab-ABD (n = 6). $^{177}$Lu-DOTA-Fab-ABD alone maintained hematologic indices largely within the normal physiological range and remained comparable to saline and trastuzumab groups. In contrast, T-DXd treatment caused marked reductions in WBC, consistent with drug-induced cytopenia. Combination therapy preserved most hematological parameters, indicating no additive hematologic toxicity relative to monotherapies.

These results demonstrate that $^{177}$Lu-DOTA-Fab-ABD exhibits a more favorable hematologic safety profile than T-DXd and retains tolerability when combined with trastuzumab, supporting its translational potential as a safer HER2-targeted therapeutic strategy.

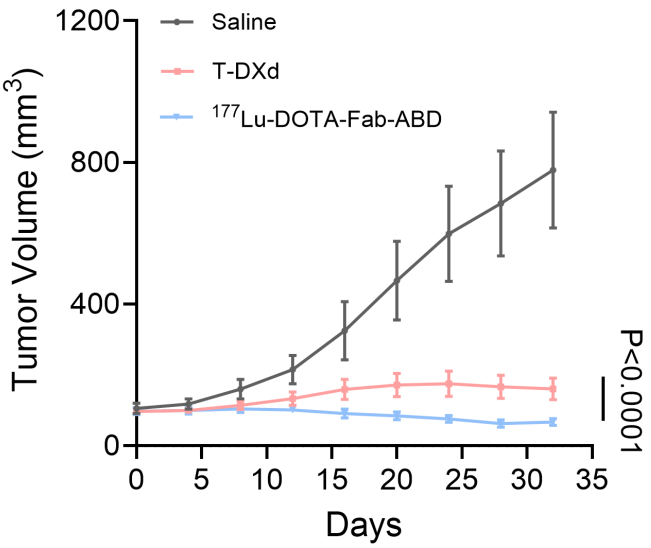


**Fig. S28. Therapeutic efficacy of $^{177}$Lu-DOTA-Fab-ABD in SKOV3 tumor-bearing mice with endogenous high HER2 expression.** Tumor growth curves of SKOV3 xenografts following treatment with saline, T-DXd, or $^{177}$Lu-DOTA-Fab-ABD. SKOV3 is a human ovarian cancer cell line with naturally high HER2 expression, without artificial genetic transfection. Mice treated with $^{177}$Lu-DOTA-Fab-ABD exhibited marked and sustained tumor growth inhibition compared with both saline and T-DXd groups. Quantitative analysis demonstrated a statistically significant reduction in tumor volume in the $^{177}$Lu-DOTA-Fab-ABD group relative to T-DXd group ($P < 0.0001$). Data are presented as mean ± SD.

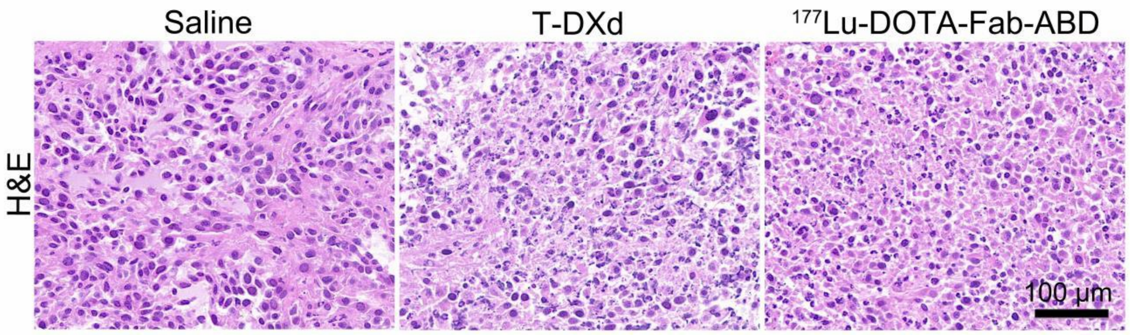

**Fig. S29. Representative H&E staining of tumor sections from saline, T-DXd, and $^{177}$Lu-DOTA-Fab-ABD groups.** H&E-stained tumor sections from mice treated with saline, T-DXd, or $^{177}$Lu-DOTA-Fab-ABD show preserved overall tumor architecture across groups. Both T-DXd and $^{177}$Lu-DOTA-Fab-ABD treatment induced notable nuclear damage and increased apoptotic features compared with saline controls, consistent with enhanced therapeutic activity. Scale bar, 100 µm.

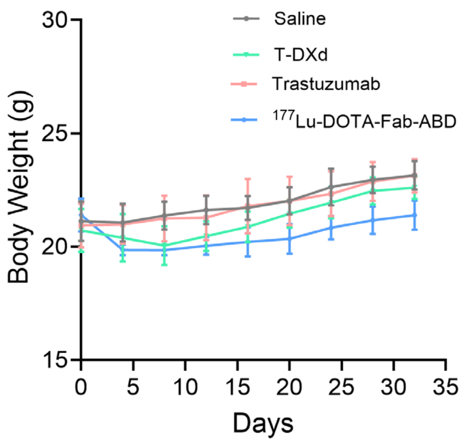


**Fig. S30. Body weight monitoring in JIMT-1 tumor-bearing mice following different treatments.** Body weight changes were monitored for 32 days after administration of saline, T-DXd, trastuzumab, or $^{177}$Lu-DOTA-Fab-ABD. All treatment groups maintained relatively stable body weights without significant loss throughout the observation period, indicating manageable systemic toxicity and good overall tolerability (n = 6).

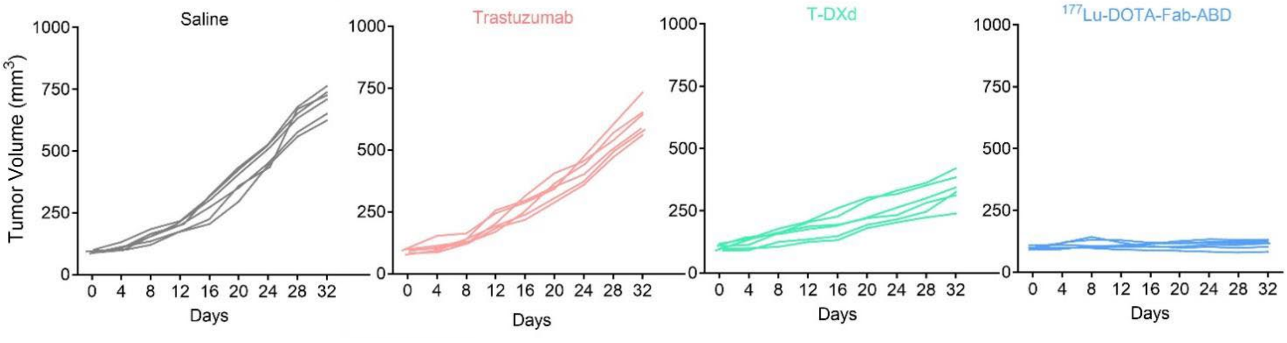

**Fig. S31. Tumor growth curves of JIMT-1 xenografts following different treatments.** JIMT-1 tumor-bearing mice were randomized into four groups (n = 6 per group) and treated with saline, trastuzumab (10 mg/kg, i.p.), T-DXd (10 mg/kg, i.p.), or $^{177}$Lu-DOTA-Fab-ABD (11.1 MBq, i.v.). Tumor growth was monitored for up to 32 days. Both T-DXd and $^{177}$Lu-DOTA-Fab-ABD produced marked inhibition of tumor progression compared with saline and trastuzumab monotherapy, demonstrating superior efficacy in this trastuzumab-resistant model.

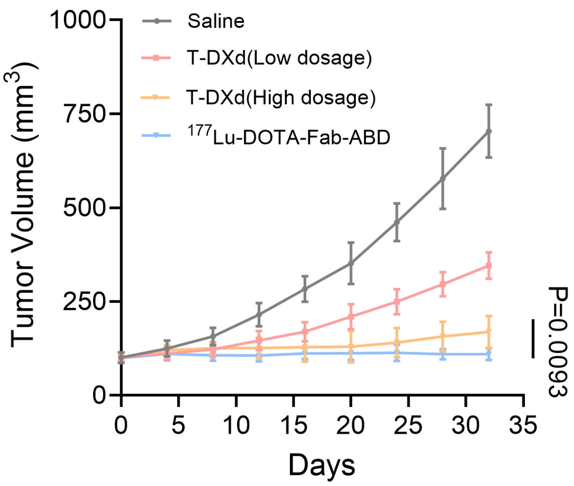


**Fig. S32. Therapeutic efficacy of $^{177}$Lu-DOTA-Fab-ABD compared with dose-escalated T-DXd in JIMT-1 tumor-bearing mice.** Tumor growth curves of JIMT-1 xenografts following treatment with saline, T-DXd at 5.4 mg kg$^{-1}$ (i.v., Q3W × 2; administered on days 12 and 33), T-DXd at 10 mg kg$^{-1}$ (i.v., Q3W × 2; administered on days 12 and 33), or $^{177}$Lu-DOTA-Fab-ABD. Escalation of T-DXd from 5.4 to 10 mg kg$^{-1}$ resulted in a marked improvement in tumor growth inhibition compared with the low-dose regimen. However, despite this dose intensification, tumor suppression in the high-dose T-DXd group remained inferior to that achieved with $^{177}$Lu-DOTA-Fab-ABD. In contrast, $^{177}$Lu-DOTA-Fab-ABD induced sustained tumor growth control throughout the observation period. Statistical analysis demonstrated significant differences among treatment groups ($P = 0.0093$). Data are presented as mean ± SD.

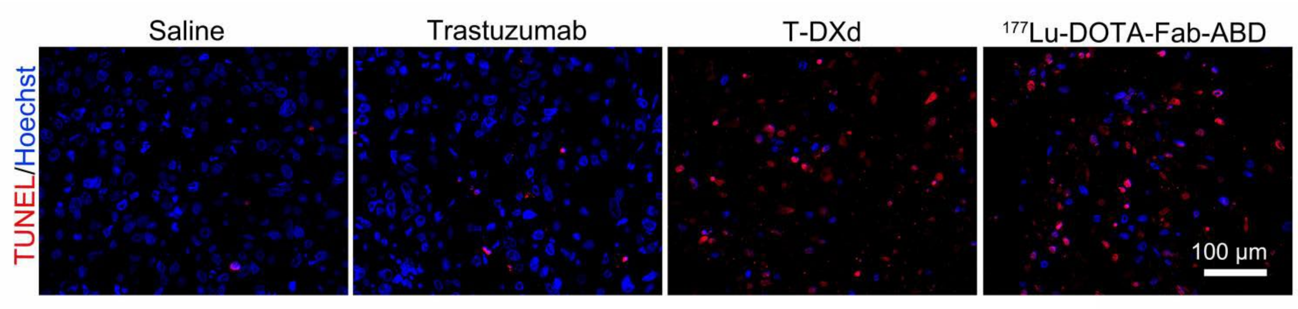


**Fig. S33. TUNEL staining of JIMT-1 tumor sections from saline, trastuzumab, T-DXd, and $^{177}$Lu-DOTA-Fab-ABD groups.** TUNEL staining showed apoptotic cells (red, TUNEL$^{+}$) and cell nuclei (blue, Hoechst) in tumor sections from mice treated with saline, trastuzumab, T-DXd, or $^{177}$Lu-DOTA-Fab-ABD. Both T-DXd and $^{177}$Lu-DOTA-Fab-ABD induced substantially higher levels of apoptosis compared with saline and trastuzumab groups. Scale bar, 100 μm.

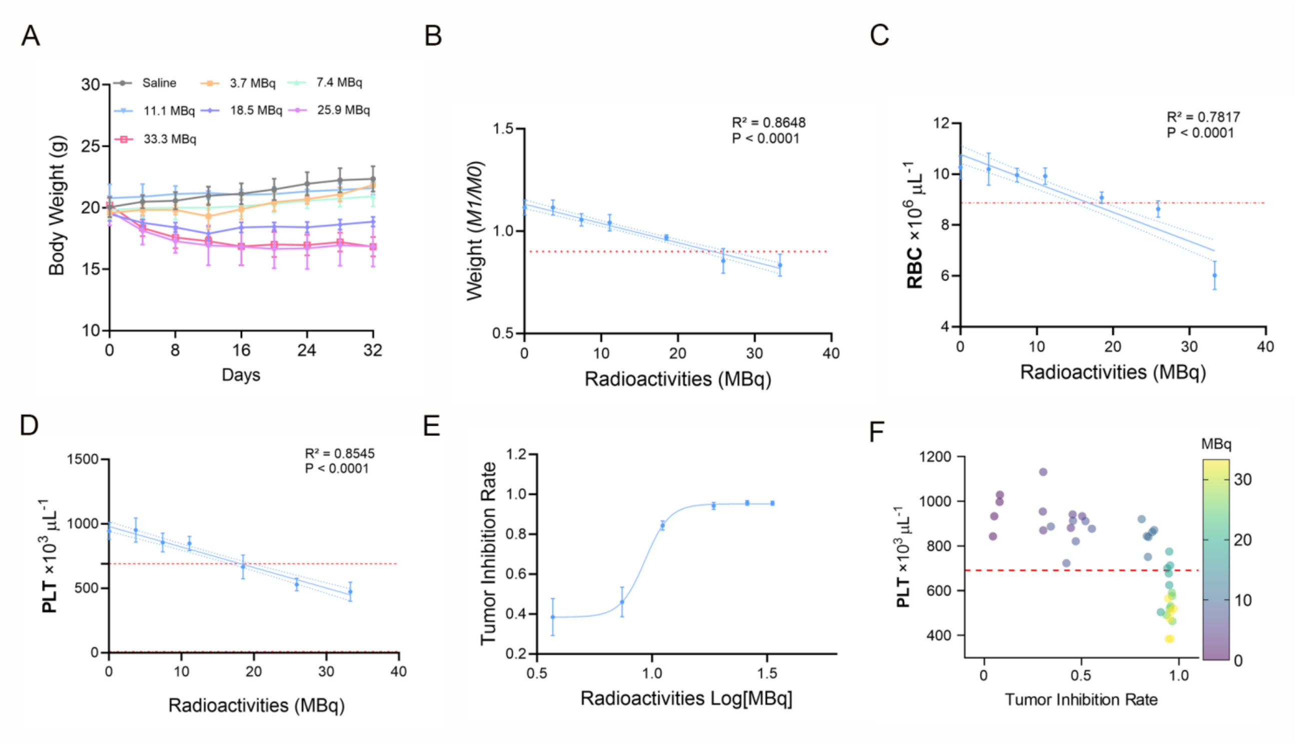


**Fig. S34. Dose-response and hematological toxicity analysis of $^{177}$Lu-DOTA-Fab-ABD in tumor-bearing mice.** (**A**) Longitudinal body weight changes in mice treated with saline or escalating activities of $^{177}$Lu-DOTA-Fab-ABD

(3.7, 7.4, 11.1, 18.5, 25.9, and 33.3 MBq). Body weight remained stable at lower activities, whereas higher activities were associated with progressive weight loss. **(B)** Correlation between administered radioactivity and normalized body weight (M1/M0). A significant negative linear relationship was observed ($R^2 = 0.8648$, $P < 0.0001$), indicating dose-dependent systemic toxicity. The red dashed line represents the predefined lower limit of acceptable body weight. (**C**) Dose-dependent reduction in red blood cell (RBC) counts. Increasing radioactivity was significantly associated with decreased RBC levels ($R^2 = 0.7817$, $P < 0.0001$). The dashed line indicates the lower physiological reference limit. (**D**) Dose-dependent decrease in platelet (PLT) counts. A strong negative correlation between administered activity and PLT levels was observed ($R^2 = 0.8545$, $P < 0.0001$), consistent with bone marrow suppression as a dose-limiting toxicity. The dashed line indicates the lower physiological threshold. (**E**) Relationship between tumor inhibition rate and log-transformed radioactivity: Tumor inhibition rate increased nonlinearly with log-transformed administered activity, displaying a typical sigmoidal dose-response pattern. Tumor inhibition rose steeply with increasing activity and approached a plateau around 18.5 MBq. (**F**) Integrated efficacy-toxicity relationship analysis. Scatter plot showing the association between tumor inhibition rate and platelet counts across activity levels, with color coding representing administered radioactivity (MBq). Higher activities achieve greater tumor inhibition but are accompanied by increased hematological toxicity, illustrating the therapeutic window of $^{177}$Lu-DOTA-Fab-ABD. Data are presented as mean ± SD where applicable.

With increasing activity, tumor inhibition exhibited a nonlinear dose-response pattern (Fig. S34E), approaching a plateau around 18.5 MBq. Beyond this intermediate dose range, further activity escalation resulted in only modest incremental gains in tumor suppression, suggesting diminishing returns in efficacy. In parallel, toxicity was evaluated using body weight (Fig. S34A, B), red blood cells (RBC; Fig. S34C), and platelets (PLT; Fig. S34D). As shown in Fig. S34A, higher activity groups

displayed more pronounced body weight reduction, and Fig. S34B demonstrates a significant inverse correlation between weight and administered activity. Hematologic parameters showed dose-dependent declines, with both RBC (Fig. S34C) and PLT (Fig. S34D) decreasing as activity increased. Among these, PLT exhibited the most sensitive and consistent response. PLT counts demonstrated a stable and significant linear decline with increasing activity ($R^2 = 0.85$, $P < 0.0001$; Fig. S34D), and began to approach and fall below the physiological threshold at and above 18.5 MBq.

Given that bone marrow suppression represents a principal dose-limiting toxicity in internal radionuclide therapy, and that platelets are generally more sensitive to radiation-induced injury, PLT was selected as the primary toxicity indicator for integrated efficacy-toxicity analysis *(33)*. As illustrated in Fig. S34F, when tumor inhibition is plotted against PLT levels, a structural divergence becomes apparent: within the intermediate dose range, tumor suppression approaches maximal levels while platelet counts remain near the physiological boundary; however, at higher activities, tumor inhibition increases only marginally whereas PLT declines substantially below the normal range. This pattern indicates progressive compression of the therapeutic window at elevated dose levels.

Taken together, the integrated analysis across Fig. S34A-F allows identification of a minimum effective dose (MED) range in which substantial tumor inhibition is achieved while hematologic toxicity remains relatively manageable. Beyond this range, further dose escalation provides limited additional efficacy at the expense of increased hematologic risk. These expanded data establish a quantitative efficacy-toxicity framework that may better inform translational dose selection for future clinical development. In clinical practice, if greater therapeutic intensity is desired, it may be theoretically feasible to incorporate hematologic supportive care or combine with other antitumor

strategies, rather than relying solely on dose escalation, in order to mitigate toxicity risk.
